# Amygdala Allostasis and Early Life Adversity: Considering Excitotoxicity and Inescapability in the Sequelae of Stress

**DOI:** 10.3389/fnhum.2021.624705

**Published:** 2021-06-01

**Authors:** Jamie L. Hanson, Brendon M. Nacewicz

**Affiliations:** ^1^Department of Psychology, University of Pittsburgh, Pittsburgh, PA, United States; ^2^Department of Psychiatry, University of Wisconsin-Madison, Madison, WI, United States

**Keywords:** amygdala, stress, neurodevelopment, brain, adversity, development, allostatic load

## Abstract

Early life adversity (ELA), such as child maltreatment or child poverty, engenders problems with emotional and behavioral regulation. In the quest to understand the neurobiological sequelae and mechanisms of risk, the amygdala has been of major focus. While the basic functions of this region make it a strong candidate for understanding the multiple mental health issues common after ELA, extant literature is marked by profound inconsistencies, with reports of larger, smaller, and no differences in regional volumes of this area. We believe integrative models of stress neurodevelopment, grounded in “*allostatic load*,” will help resolve inconsistencies in the impact of ELA on the amygdala. In this review, we attempt to connect past research studies to new findings with animal models of cellular and neurotransmitter mediators of stress buffering to extreme fear generalization onto testable research and clinical concepts. Drawing on the greater impact of inescapability over unpredictability in animal models, we propose a mechanism by which ELA aggravates an exhaustive cycle of amygdala expansion and subsequent toxic-metabolic damage. We connect this neurobiological sequela to psychosocial mal/adaptation after ELA, bridging to behavioral studies of attachment, emotion processing, and social functioning. Lastly, we conclude this review by proposing a multitude of future directions in preclinical work and studies of humans that suffered ELA.

## Introduction

The amygdala has been the focus of a great deal of attention in research aimed at understanding the effects of Early Life Adversity (ELA). The fact that this evolutionarily ancient brain structure is of interest is perhaps not surprising given this region's essential role in socioemotional functioning (Bachevalier et al., 1999; Amaral, 2002) and that forms of ELA (e.g., child abuse; child neglect) engender problems with regulating the emotions and behaviors (Kessler et al., 2010). In this review, we first discuss the basic functions and development of this brain region, noting why this area has been a strong candidate for understanding the multiple mental health issues common after ELA. We next explore past research focused on this brain region in human and preclinical models of ELA exposure and potential of an allostatic load model to disentangle apparent inconsistencies in these findings. We then extend this idea, pulling from parallel models put forth in research studies focused on autism and neurodevelopment, and integrating preclinical rodent and nonhuman primate findings, to make specific hypotheses about human behavioral and clinical correlates of specific cellular and neurotransmitter changes. We finally close this document with proposals for future research directions connected to these ideas.

## Defining Early Life Adversity and Reviewing Connections Between ELA and Poor Mental Health

Surveying work on ELA, researchers have focused on different samples exposed to adversity including child maltreatment (e.g., physical or sexual abuse), extreme household dysfunction (e.g., having a parent with a severe mental illness), and poverty (alongside lower “social standing”). These and related negative experiences have been referred to using different umbrella terms, such as “early life stress,” “child trauma,” “toxic stress,” “early adversity,” and “Adverse Childhood Experiences (ACEs).” An important starting question is whether to lump adversities together, think about specific experiences (e.g., early social neglect or physical abuse), or examine potential shared dimensions of ELAs. Initial research took a purely cumulative exposure approach, summing up the total number of adversities suffered, or looking at child trauma *across* different forms of maltreatment (Felitti et al., 1998; Hanson et al., 2012a; Gorka et al., 2014). This approach has high explanatory power and can deal with the common pattern of co-occurrence between many forms of adversity (Appel and Holden, 1998; Emery and Laumann-Billings, 1998; Kellogg and Menard, 2003); however, cumulative models provide less clarity about potential mediating mechanisms. More recently, starting frameworks (Belsky et al., 2012; McLaughlin et al., 2014a) argue for the difference between dimensions of adversity (i.e., harshness vs. unpredictability; deprivation vs. threat) to advance mechanistic understanding of the impact of ELA. Moving forward, the field must strike a balance between more mechanistic approaches and the reality of the high co-occurrence of different ELAs, as well as the low-base rates for an isolated form of adversity. In this space, there are multiple reviews about this topic (e.g., McLaughlin et al., 2020; Smith and Pollak, 2020) and we would direct readers to those past publications for more in-depth discussion. Here, we take a more broad and inclusive definition of ELA. This is in keeping with many preclinical approaches and the well-known ACEs study from the Centers for Disease Control and Prevention. These experiences share some core elements in that they can be psychosocial hazards, are severe deviations from the expected environment, and activate stress responsive physiology (as thoughtfully discussed by Nelson and Gabard-Durnam, 2020). We, however, return to this issue in later sections of this document.

While definitions are variable, clear from a large body of research is that multiple forms of ELA are associated with compromised development and long-term physical and mental health challenges (Shonkoff et al., 2012). Across different models and forms of ELA, a rigorous body of work has established a strong connection between these experiences and antisocial and aggressive behavior (or so-called “*externalizing psychopathology*”). For example, greater disruptive behavior and conduct problems have been found in victims of child sexual abuse (Mallett and Schall, 2019), in individuals who suffer physical abuse or neglect (Moylan et al., 2010; Muniz et al., 2019), and in youth from households with lower income (Votruba-Drzal, 2006; Evans and Cassells, 2014; Piotrowska et al., 2015). Turning to depression, anxiety, and other forms of “*internalizing*” psychopathology, similar patterns have been noted, with major depressive disorder (MDD) being associated with child maltreatment (Nanni et al., 2012; Björkenstam et al., 2017) and to a lesser, though still significant, extent after exposure to poverty (Letourneau et al., 2013; Peverill et al., 2020). ELAs are often associated with a more severe and chronic course of MDD (Chapman et al., 2004; Wiersma et al., 2009; McLaughlin et al., 2011; Carr et al., 2013), as well as poorer response and remission outcomes for the treatment of this disorder (Williams et al., 2016). Examined collectively, research has consistently linked ELA with a plethora of negative mental health outcomes, with risk commonly increasing with each additional exposure (Felitti et al., 1998). Understanding the potential mechanisms by which ELA worsens mental health, as well as candidate mechanisms of resilience and recovery, is critical to prevention, intervention, and ultimately curative treatments.

## The Amygdala as an Important Socioemotional Hub: Consideration of Basic Functions and Neurodevelopment

Situated in the anterior portion of the temporal lobe, the amygdala is a complex of subcortical nuclei important for the evaluation of the emotional significance of incoming stimuli (Davis and Whalen, 2001). While the constituent amygdala subnuclei each subserve different functions (*described later in this document*), collectively the amygdala calculates the intensity of response to positive and negative emotional stimuli (Ambroggi et al., 2008; Fox et al., 2015). Because of its connections to evaluative regions in frontal cortex, contextual information from hippocampus, procedural and reward information from striatum, and autonomic outputs to the hypothalamus and ascending cholinergic nuclei, the amygdala can mediate adaptive physiological (e.g., autonomic reactivity) and behavioral (e.g., reallocation of attentional resources) responses to varied environmental and social challenges (Phelps, 2004; Hariri, 2009). In line with these ideas and its involvement in fear learning, meta-analyses of functional neuroimaging studies in humans find the amygdala is activated by a number of negative emotions (Lindquist et al., 2012), with direct stimulation of human amygdala confirming the primacy of fear and anxiety (Lanteaume et al., 2007).

Given these basic functions, research focused on different forms of psychopathology have centered on the amygdala. Various mood and anxiety disorders [e.g., MDD; generalized anxiety disorder (GAD); post-traumatic stress disorder (PTSD)] and some samples with autism have shown greater amygdala *activation* to facial displays of fear and anger (Etkin and Wager, 2007; Hamilton et al., 2012). Differences in amygdala *structure* have also been noted in individuals with excessive socioemotional responses ranging from autism (Nacewicz et al., 2006; Kim et al., 2010), to MDD, to social phobia to PTSD (Karl et al., 2006; Woon and Hedges, 2009). Examined collectively, these different bodies of research underscore the amygdala as central to emotion processing, with aberrant structure and activity in multiple forms of psychopathology.

Thinking about the amygdala and neurodevelopment, it is important to note that nuanced work has begun to illustrate that the typical development of the amygdala is non-linear in nature, similar to overall cortical development (Shaw et al., 2006), with amygdala development continuing well into adulthood. Substantial post-natal development may mean that environmental experience has a greater potential to significantly impact and influence neurodevelopment. The basic structural architecture of the amygdala is well-established at birth, but volumes increase significantly during infancy (Humphrey, 1968; Ulfig et al., 2003). Though some cross-sectional reports suggest a general decrease in volume during adolescence and in early adulthood, longitudinal quantitative MRI work (Wierenga et al., 2014), as well as analyses focused on amygdala histology (Cunningham et al., 2002; Saul et al., 2014), suggest a more complex pattern. This work indicates amygdala volumes *relative to brain volume* continue to increase through adolescence, reaching maximum volumes in the late teens or early twenties. The exact age of these peaks is, however, dependent on the sex and pubertal dynamics of an individual (Goddings et al., 2014). Such trajectories fit with preclinical work finding active periods of cell proliferation in these regions during adolescence (Saul et al., 2014; Sorrells et al., 2019) and continued development in human post-mortem studies (Avino et al., 2018)

In sum, research underscores that the amygdala is central to emotion processing, and its abnormal structure and predominantly excessive activity are common to different forms of psychopathology. Furthermore, the amygdala displays rapid structural growth early in life, with continued refinement of this anatomy into adolescence and early adulthood. Variations in outcomes, both behaviorally and neurally, may be due to ELA impinging upon core developmental processes happening at the specific time of stress exposure. These core functions and neurodevelopmental trajectories are important bedrocks to consider when thinking about the effects of ELA on amygdala structure and potential critical periods for important affective processes, such as the buffering against generalization of fear.

## What is the State-Of-Science of Adversity'S Impact of the Amygdala? What Might Be Causing These Inconsistencies?

Surveying preclinical research, as well as studies in human samples, it is clear that stress exposure and exposure to adverse experience impacts amygdala structure; however, the magnitude and directionality of these effects has been challenging to understand and to cohesively summate. In regards to preclinical work, these studies, primarily conducted in late juvenile or early adult rodents, has found exposure especially to restraint stress leads to volumetric *increases* such as dendritic arborization in amygdala nuclei (Vyas et al., 2002; Mitra et al., 2005; Cohen et al., 2013); this is opposite the hippocampal changes where dendritic retraction is typically seen after stress (Watanabe et al., 1992; Magariños et al., 1996, 1997). Initial work in human adults did not find alterations in amygdala structure in samples exposed to ELA (Bremner et al., 1997; Cohen et al., 2006). A recent study, however, noted larger amygdala volumes in adults who were exposed to higher levels of cumulative stress during childhood (Evans et al., 2016). This was in contrast to a large study of non-demented older adults (*N* = 466) that found participants who reported two or more early-life events had significantly smaller amygdalae with increasing age (Gerritsen et al., 2015). When adults had a history of ELA exposure and comorbid psychopathology (such as PTSD or borderline personality disorder), smaller amygdala volumes have typically been reported in ELA-exposed samples (Driessen et al., 2000; Schmahl et al., 2003; Weniger et al., 2008; Irle et al., 2009; Veer et al., 2015; Souza-Queiroz et al., 2016). However, in a unique sample of adolescents and adults with elevated risk for psychosis, no associations between adversity and amygdala volumes were found (LoPilato et al., 2019).

Structural neuroimaging in human pediatric populations have, similarly, yielded mixed results. In children exposed to neglect, research reports have noted larger amygdalae (Mehta et al., 2009; Tottenham et al., 2010; Roth et al., 2018), as well as no differences (Sheridan et al., 2012; McLaughlin et al., 2014b; Hodel et al., 2015). Child poverty has been associated with larger (Noble et al., 2012) and with smaller amygdalae (Luby et al., 2013; Ellwood-Lowe et al., 2018). Smaller amygdalae (Edmiston et al., 2011; McLaughlin et al., 2016), as well as no differences, have been found in adolescents who experienced child maltreatment (De Bellis et al., 1999, 2001, 2002; Carrion et al., 2001; Gold et al., 2016). Similarly, exposure to community violence during childhood was related to smaller amygdala volumes (Saxbe et al., 2018; Weissman et al., 2020); however, related recent work did not replicate this association in a similar sample (Butler et al., 2018).

Our research group attempted to deal with some of these inconsistencies by using a rigorous tracing protocol and focusing on three different forms of ELA—child poverty, physical abuse, and early social neglect—in a sample of youth ages 9–14. This work also deployed rich measures of stress exposure, obtained through semi-structured interviews with both youth and parents. Interestingly, while reduced amygdala volumes were common to all types of ELA and not statistically differentiable at our sample size, the impact of low SES was greatest with physical abuse slightly worse than institutional neglect (Hanson et al., 2015b). A portion of these differences could reflect our finding that greater cumulative stress exposure was associated with smaller amygdala volumes. Recently, Herzog et al. (2020) tried to compare the impact of different types of ELAs using cutting-edge statistical methods (random forest regression) and found neglect during childhood and adolescence was related to smaller amygdala volumes. However, work using latent class models to identify classes of ELA (e.g., Family Instability; Direct Victimization) did not find any associations between ELA type(s) and amygdala volume (King et al., 2019). Future studies with large samples that *are equally matched on stress severity* could potentially differentiate unique contributions of ELA type.

Across these studies, one major limitation is that the preponderance of this work has been cross-sectional in nature. Such work can miss the complexity of neurobiological trajectories, underscoring the importance of studying development longitudinally (Shaw et al., 2006; Wierenga et al., 2014). In regard to longitudinal samples, Whittle et al. (2013) found childhood maltreatment was associated with slower growth of the left amygdala, but these associations reversed if participants presented with psychopathology. Mirroring some of these patterns, VanTieghem et al. (2021) used an accelerated longitudinal design to compare youth who previously suffered early social neglect in institutional care and a comparison sample without such ELA. Youth who suffered early social neglect had a reduced growth rate of the amygdala, resulting in smaller volumes by adolescence. Given the panoply of inconsistent findings reviewed here, it will be important to judiciously walk through potential sources of measurement error and biological/physiological variance as the field continues to think about connections between ELA and neurodevelopment.

Surveying the human neuroimaging studies focused on ELA, there is a wide-range of variation in methodology, sampling strategies, and conceptualizations of ELA and related stress. Each of these areas likely interjects inaccuracies and biases in reported results. At a basic level, volumetric quantification of the amygdala is more complex, and potentially inaccurate, than many may allude to. Volumetric amygdala measurement can be performed using manual and automated protocols. A good deal of the early structural imaging work employed manual tracing of the amygdala; this methodology can often be more precise and accurate, but is time-consuming and requires extensive expertise. In our own work at the University of Wisconsin-Madison, even with 8 months of training, 80–90% of undergraduate trainees failed to reach spatial and numerical reliability on our whole amygdala segmentation, and expert tracing still requires at least 2 h per amygdala (e.g., Caldwell et al., 2015). This is now amplified by a factor of six or more, as we hand-trace individual subdivisions and subnuclei. Manual tracing can still be problematic if poorly executed, evidenced by over constraint (i.e., highly precise but insensitive to individual variation) or simple drift in technique leading to high variability (e.g., low intraclass correlation coefficients). For example, Cohen et al. (2006) reported that the average amygdala volume for an adversity-exposed group was 1.27 and 1.16 mL (for the right and left amygdala), while a non-adversity exposed group was 1.26 and 1.15 mL (for the right and left amygdala). These groups were not significantly different from one another. However, rarely highlighted is that the error for these measures was actually higher than the mean volumes (ELA group = 1.40 for right, 1.28 for the left; Comparison group = 1.43 for right, 1.36 for the left). This suggests inconsistent and problematic hand-tracing, and similar results (*null or otherwise*) with these patterns should likely be greeted with skepticism. Moving away from hand-tracing, there are now many commonly available automated methods for amygdala volumetric quantification (e.g., Hanson et al., 2012b; Buser et al., 2020; Liu et al., 2020). These approaches represent a scalable and easy-to-deploy method to potentially test relations between volumetric measures and psychological variables of interest; such methods may be particularly important given that structural MRI-datasets are exponentially increasing in size (from 10 to 1,000 s). However, many approaches (i.e., *Freesurfer*) often yield unsatisfactory results with high-variability and low-validity (Babalola et al., 2009; Morey et al., 2009; Dewey et al., 2010; Hanson et al., 2012b). For example, we found that automated segments of the amygdala generated by Freesurfer had low bivariate correlations with volumes from rigorous hand-tracing of the same structure (Left *r* = 0.563, Right: *r* = 0.560; (Hanson et al., 2012b). Particularly damning, in Hanson et al. (2015b), Freesurfer-estimated amygdala volumes captured neither group differences nor individual differences in cumulative life stress in a sizable sample of youth who suffered different forms of ELA

For high-throughput studies, a new generation of automated segmentation tools is required. In our recently published approach (Liu et al., 2020), accurate amygdala acquisition and segmentation required modifications in both a Multi-Atlas model and a Convolutional Neural Network. Multi-Atlas models match overall context, as cost is calculated across the whole brain, but requires hyperbolic exaggeration of subtle boundaries to distinguish the amygdala subnuclei. In contrast, the neural network easily matches fine details, but requires combination with a parallel network constraining the model on a larger contextual scale. Either of these dual-scale approaches is acceptable, but all segmentations require additional visual quality checks.

Turning to issues with study designs, many investigations in humans have had a large age range of participants (e.g., 5–15 years old in studies focused on pediatric populations); this is particularly important to note given amygdala developmental trajectories reviewed earlier. For example, LoPilato et al. (2019) examined a large cohort of individuals, but the age range spanned from 12 to 30 years of age. During this span, amygdala structure is actually increasing in volume, hitting a peak volume, and possibly shrinking again; mixing of age groups likely occludes associations between ELA and volume. Connected to this, in most work, age is simply added as a linear covariate to statistical models. Research might think of alternative strategies for studies where participants span multiple developmental epochs (or large age ranges, i.e., individually fitting a quadratic term of age). For instance, Merz et al. (2018) examine the interaction of age and family income/poverty, one type of ELA, in a sizable cohort of youth (*N* = 296). When these investigators examined the full cohort, there were no significant effects detected. But, looking at age X ELA interactions, these investigators found that lower family income was significantly associated with smaller amygdala volumes in adolescence (13–21 years old). However, this relation was not seen for younger age children (3–12 years), suggesting important neurodevelopmental associations may only be revealed when considering ELA and developmental stage(s).

We believe that these confusing results can be explained by the inverted-*U* allostatic growth trajectory. High levels of stress initially increase amygdala volume, but the most extreme (or chronic) levels of adversity may result in smaller volumes. Support for this idea comes from multiple avenues. First, cross-sectional studies suggest complex associations between amygdala structure, the intensity of ELA, and developmental consequences of stress. For example, Mehta et al. (2009) found larger amygdalae in children exposed to early social neglect; however, the duration of early neglect (that these same children were exposed to) was actually related to smaller amygdalae. Similarly, combat-exposed adults with PTSD exhibited larger amygdalae compared with their non-PTSD counterparts. But, in individuals with a history of ELA and PTSD, smaller amygdala volumes were actually found (Kuo et al., 2012). A recent multi-group study by Morey et al. (2016) that examined maltreated youth with PTSD, without PTSD, and non-maltreated healthy volunteers further highlights this. Maltreated youth without PTSD demonstrated larger amygdalae compared with maltreated youth with PTSD and compared with non-maltreated control youth. However, PTSD symptoms were correlated with amygdala volumes, with greater symptomatology being related to smaller volumes.

**Figure 1:**
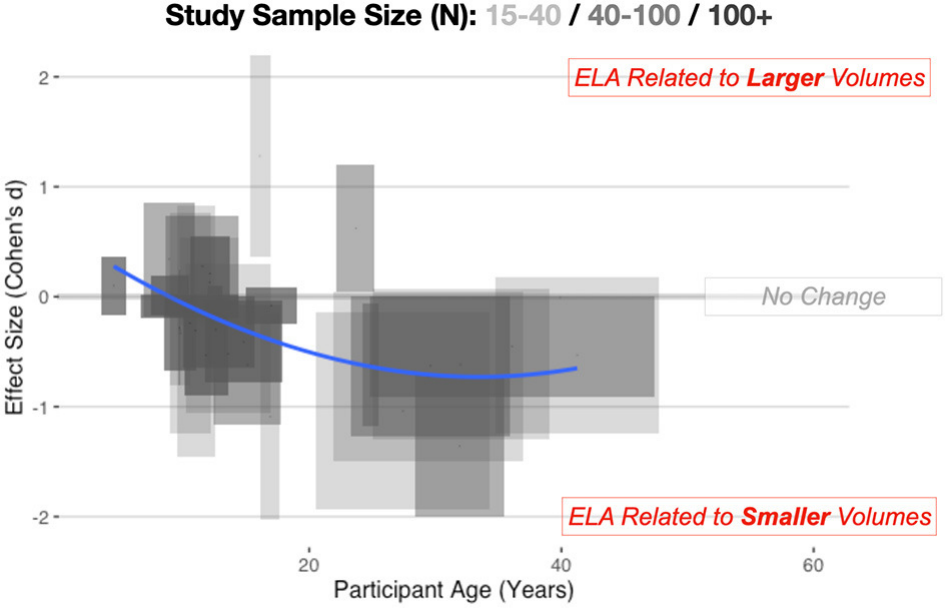
Combining across automated and manual methods for quantifying the amygdala, here we depict amygdala volumetric differences for ELA-exposed samples. Cohen's d, with 95% confidence intervals (CIs) of effects, are shown on the vertical axis, while the span in age is shown on the horizontal axis. Longer boxes (on the horizontal axis) indicate studies with larger age-ranges in their samples, while wider boxes (on the vertical axis) depict studies where the effect size estimates and 95% CIs span a larger numeric range. Study sample size is also depicted in this graphic with lighter boxes being studies with smaller sample sizes and darker boxes representing studies with a larger number of participants. Individual study data is available online at: https://github.com/jlhanson5/Hanson_Nacewicz_Frontiers_Amygdala_Review_Data.

This pattern is visibly evident looking across multiple studies (Figure 1) considering age of the sample and the direction of effect. For younger samples (<9 years of age), there is reasonable data to show volumetric increases in the amygdala, but looking at adult samples, there is the suggestion of smaller volumes. In past meta-analyses, there has often been aggregation of different studies but limited consideration of a non-linear trajectory. This is perhaps why there has been conflicting results across different meta-analyses. Taking a more thoughtful developmental perspective supports this inverted-*U* pattern of alterations. For example, recent longitudinal work (Whittle et al., 2013) suggest a slowing of growth of the amygdala after ELA. Particularly important to highlight, youth who suffered early social neglect, one form of ELA, had a reduced growth rate of the amygdala, resulting in smaller volumes by adolescence (VanTieghem et al., 2021) and it is as-yet unknown if this represents a delay or a missed critical period to learn fear and safety.

Looking at ELA as a form of allostasis raises many testable questions. Consider the measurement and definition of ELA. These concepts are notoriously difficult to measure and may take many forms. For example, in samples exposed to poverty, in addition to challenges with low income, there are often greater residential neighborhood problems in impoverished environments (Steptoe and Feldman, 2001). Higher crime, inadequate neighborhood services, and transportation problems may constitute sources of chronic stress. There are also more daily “mundane” stressors in low SES environments and this may contribute to greater rates of psychopathology (Kanner et al., 1981; Almeida, 2005; Odgers and Jaffee, 2013). Indeed, as Slavich (2019) noted the large preponderance of life stress exposure work is “*measuring only the superficial contours of this complex construct*.” There is a massive and significant variation in severity, frequency, timing, and duration of adversity, and as yet the relative weight of these against disruptions of parental attachment is unknown. Each of these factors could likely be introducing heterogeneity in the large body of findings we reviewed above. As we discuss later in this document, it is likely that different forms of ELA may share phenomenological elements (e.g., experiences of threat; McLaughlin et al., 2014a).

## “Amygdala Allostasis:” Focusing on Excitatory/Inhibitory Neurochemistry and Considering Behavioral Consequences After ELA

**Figure 2:**
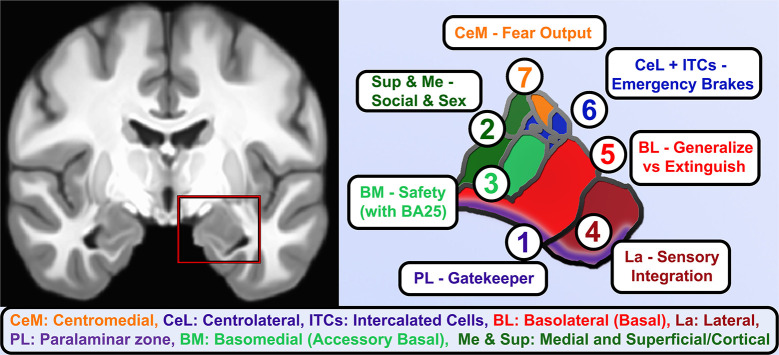
Functional roles of human amygdala subnuclei. A coronal view of T1-weighted structural MRI (left) showing amygdalae resting on the anteriormost hippocampus and separated by the white matter of the alveus, and closeup of hand-segmented amygdala subnuclei (right). (1) While there is still debate about whether the paralaminar region is a true nucleus vs. the subventricular region of other nuclei, it is the main zone of from which newborn neurons migrate into the basal and lateral nuclei and houses dopamine-innervated GABAergic cells that gate activation of the basal and lateral nuclei. (2) The corticial nucleus and superficial nuclei are closely linked to the olfactory system and in vomeronasal animals coordinate responses to pheromones. As such, these and the medial nucleus of the amygdala contribute to latent drives such as recognition of conspecifics, maternal attachment, and sex-related differences and behaviors. (3) Basomedial nucleus is an early-developing nucleus that bears some functional similarities to the adjacent superficial nuclei, e.g., changing serotonin receptor expression in studies of early maternal separation, but also brings in information about safety cues from higher centers through direct innervation by infralimbic/BA25 projections. (4) The lateral nucleus gathers information about threatening cues and contexts from highly processed sensory information and contextual information from hippocampus. It is the largest nucleus in humans and most reliably enlarges in allostatic load, consistent with rodent studies showing dendritic expansion as fear generalizes. (5) The Basolateral or simply Basal nucleus similarly expands volume and dendrites after inescapable stress, but it is also home of key “extinction cells” that integrate information from other nuclei and prefrontal inputs and feeding forward inhibition to contextualize or extinguish threat responses and turn off dopamine from the VTA. (6) The intercalated cell islands (ITCs) and about half the cells in the lateral division of the central nucleus (CeL) receive feedforward inhibition from basal nuclei or are directly activated by the social bonding hormone oxytocin (CeL), to inhibit latent and previously learned fear responses. They receive heavy dopaminergic innervation and send effectors to centromedial (CeM) and a similar lateral-to-medial inhibition of the ascending arousal signaling of the cholinergic basal nucleus of the stria terminalis. About half the neurons are so-called “fear on” neurons that signal latent and previously-learned fears with some threat signals from innervation by the paraventricular thalamus. (7) CeM sends long-range projections that tonically inhibit hypothalamic and brainstem autonomic centers until fear and safety signals integrated by CeL shifts toward threat and inhibits these neurons.

**Figure 3:**
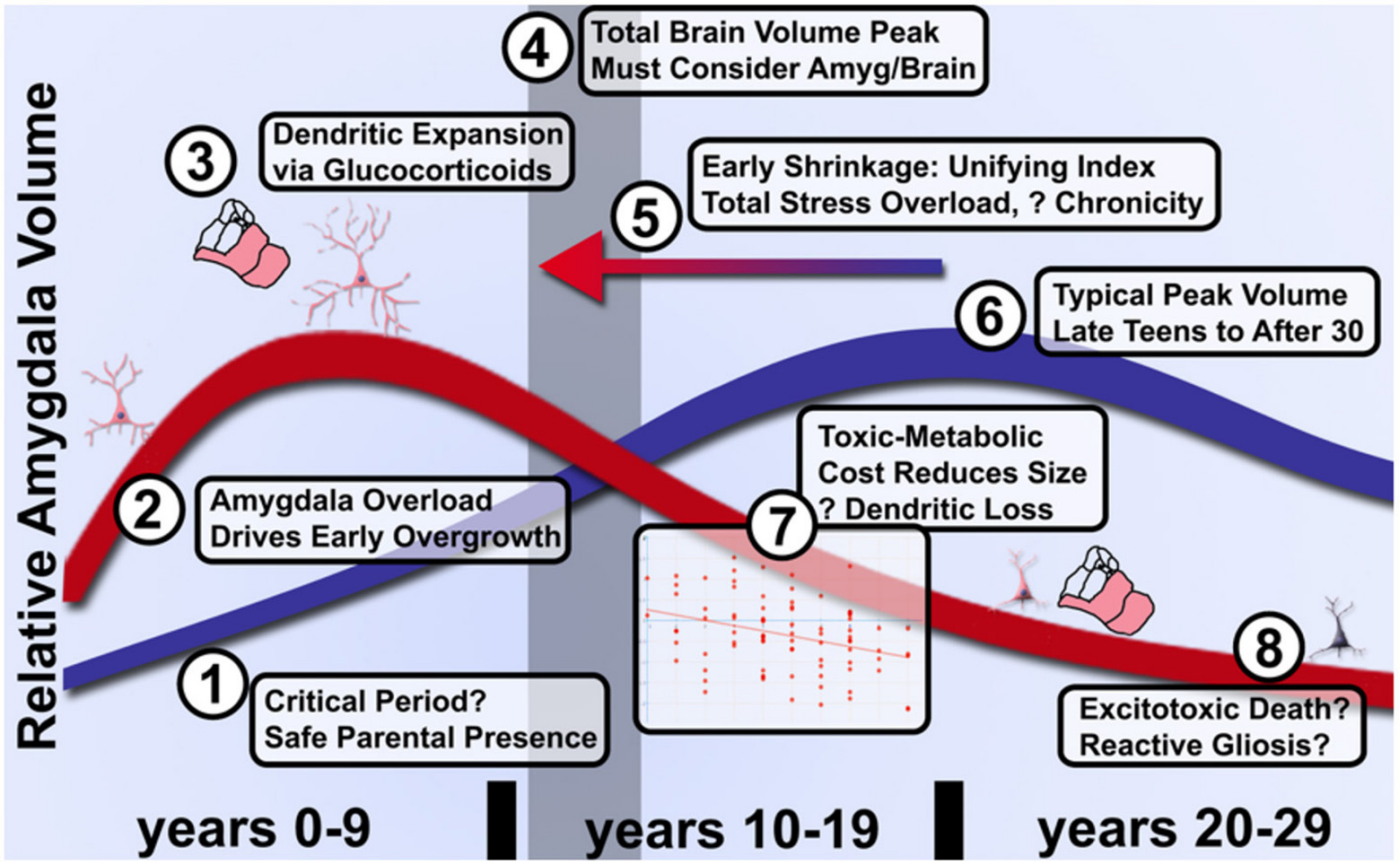
Model of graded acceleration of amygdala volume relative to brain volume and regions of clarification needed in future studies of allostatic load in ELA. (1) It has not been established whether a critical period exists during the first several months of parental attachment or after during which parental presence facilitates overcoming adversity and an enduring sense of parent-cued safety. (2) As noted above, more evidence will be needed to clarify whether early enlargement or rate of growth is quantitatively linked to degree of subjective adversity. (3) While certainly involving glucocorticoids, dendritic expansion and a shift toward excitation over inhibition, the limits on peak volume and transition to shrinkage is poorly understood even in ASD. (4) These changes relative to control volumes must be further couched in an understanding of typical brain development that likely peaks by the preteen years, thus even a shift earlier in life (5) of the same curve could manifest as a distinct quadratic shape in proportion to severity of adversity. (6) Furthermore, the peak of typical amygdala development and influences on timing have not been fully elucidated due to the challenges of longitudinal study. (7) Provided individuals are assessed after the quadratic peak, amygdala shrinkage quantitatively reflects cumulative life adversity and better characterization of this pathophysiology may distinguish effective treatments from the natural course of disease. (8) Whether inflammation in the form of reactive gliosis and excitotoxic death occur and can be prevented will require better longitudinal tools that track microsctructure and neurochemistry longitudinally.

When considering the potential neurodevelopmental impact of ELA, it is critical to realize that: (a) the amygdala is not a unitary brain area, but rather heterogenous subnuclei with unique functions and developmental trajectories (as detailed in Figure 2); and (b) stress may exert non-linear effects in concordance with McEwen's notions of “allostatic load.” Connected to heterogeneity, volumetric growth during development varies across subnuclei, but is driven by an increase in neurons of the basal nuclei and lateral nucleus. Other subnuclei of the amygdala (i.e., the paralaminar region) gradually loses putative newly generated/differentiated neurons, suggesting a proliferative role (or migration pathway) akin to subventricular zones of neighboring structures (Chareyron et al., 2012; Avino et al., 2018; Jurkowski et al., 2020). Related to allostatic, inverted-*U* patterns, allostatic load translates roughly to “a new normal” and is the detrimental physiological consequence caused by sustained excessive activation of stress-responsive systems (Danese and McEwen, 2012); this is commonly caused by chronic or repeated exposure to psychosocial stressors. With stress and ELA exposure, we specifically believe that increases in cellular complexity in the amygdala (e.g., higher dendritic branching; increased synaptogenesis) leads to excessive excitation, untamable by inhibitory synapse growth, and this cascades to excitotoxic damage and ultimately cell death (schematized in Figure 3). Connecting these two elements, we believe that basolateral portions of the amygdala largely encode cues, contexts and behaviors mapping the boundary between *safety* and known threats.

In regard to allostatic load of the amygdala and nearby structures, recent preclinical work shows that neurogenesis in hippocampus is highly constrained by the metabolic costs of deviating from an optimal ratio of excitatory to inhibitory neuronal firing (Wang et al., 2020). Considering metabolic costs of excitation-inhibition ratios in the amygdala (Figures 2, 3), the basolateral portions are primarily excitatory with a large concentration of glutamate (Glu) releasing neurons, but under resting conditions an extensive network of inhibitory, γ-aminobutyric acid (GABA) releasing neurons anchored in the paralaminar zone largely silences the basolateral complex (Quirk and Gehlert, 2003). Modulatory neurotransmitters (Marowsky et al., 2005) relieve this inhibition, bringing online the basal and lateral nuclei that compute the magnitude of feed-forward fear and safety signals and set the degree of fear generalization. Manipulating GABA in this region modulates amygdala reactivity and reduces anxiety and social behaviors (Sanders and Shekhar, 1995; Paulus et al., 2005; Del-Ben et al., 2012). Multiple forms of affective psychopathology are theorized to be related to excessive Glu-GABA ratio in this region (Sanders and Shekhar, 1995; Cortese and Phan, 2005; Pittenger et al., 2007; Tye et al., 2011). The basolateral complex ultimately sends information about conditioned and aversive stimuli to the centromedial “output” nuclei (Duvarci and Pare, 2014) which, like neighboring striatum, have among the highest density of GABA synapses in the brain (Sutoo et al., 2000). Central subnuclei integrate the ascending information with previously learned fear and social hormonal signals to ultimately trigger autonomic and behavioral fear responses through brainstem projections.

With increasing levels of chronic stress (such as in ELA), there is interruption of the normal excitation-inhibition balance in the amygdala. This may occur through multiple pathways and may explain a portion of the heterogeneity of structural results seen previously. First, stress may cause higher excitability in the basolateral amygdala, due to: increases in the number of spontaneously firing neurons (Zhang and Rosenkranz, 2012), enhanced excitatory synaptic drive (Padival et al., 2013), and the increased expression and activation of glutamatergic N-methyl-D-aspartate (NMDA) receptors (triggering so called “silent synapses”; Mozhui et al., 2010; Suvrathan et al., 2014; Tzanoulinou et al., 2014b). In addition, preclinical work indicates stress exposure can lead to GABAergic alterations. Stress during the juvenile period is related to changes in GABA-A protein expression (Jacobson-Pick and Richter-Levin, 2012; Tzanoulinou et al., 2014a) and reduction in enzymes involved with synthesis of GABA in the rodent amygdala (Tzanoulinou et al., 2014b). Interestingly, adversity during the juvenile period may actually result in an immature-like expression profile of the GABA-A receptor subunit (Jacobson-Pick et al., 2008). This may be compensatory, as stress leads to long lasting loss of tonic GABA-A receptor currents in the projection neurons of lateral amygdala (Liu et al., 2014). Finally, stress may cause alterations in cortisol, cannabinoids, and neuropeptides, such as cholecystokinin and neuropeptide Y (Shekhar et al., 2005); alterations in these systems may further indirectly impact excitation-inhibition balance in the amygdala (Hadad-Ophir et al., 2014; Radley et al., 2015). Examined collectively, stress impacts both inhibition and excitation in the amygdala through direct alterations in Glu and GABA, as well as through indirect stress-induced changes in hormonal and neuropeptide signaling. These multiple pathways tilt the amygdala to a more excitable state, paralleling the human findings of amygdala hyper-reactivity to emotional stimuli after exposure to ELA.

At a larger scale, one sees a more overall excitable amygdala, with increased dendritic spines in basolateral nuclei after adversity (Vyas et al., 2002, 2003, 2004, 2006; Mitra et al., 2005). Despite these neurobiological alterations, organisms exposed to stress must still strive to maintain homeostasis, regulating their physiological and behavioral responses to environmental experiences. This hyper-excitable state of the amygdala, however, has the potential to lead to wear and tear on the body and brain (allostatic load and overload; McEwen, 1998; McEwen et al., 2015). Thinking about these patterns, McEwen (2003, 2005) noted parallels with brain alterations in humans during initial episodes of major depression, where larger volumes and increased functional activity of the amygdala have been noted (Frodl et al., 2003). McEwen further suggested that this hyperactivity might give way to eventual shrinkage, citing reports of smaller amygdalae after repeated depressive episodes (Sheline et al., 1999). Knitting together work in stress exposed juvenile animals, one sees preliminary support for this idea. Work from Rosenkranz et al. found repeated stress increases the excitability of amygdala neurons (Hetzel and Rosenkranz, 2014), but loss of spines in the amygdala after repeated stress during early development (Padival et al., 2015). Particularly interesting, animals resilient to stress had markers of reduced excitatory drive from glutamatergic inputs in the amygdala.

**Figure 4:**
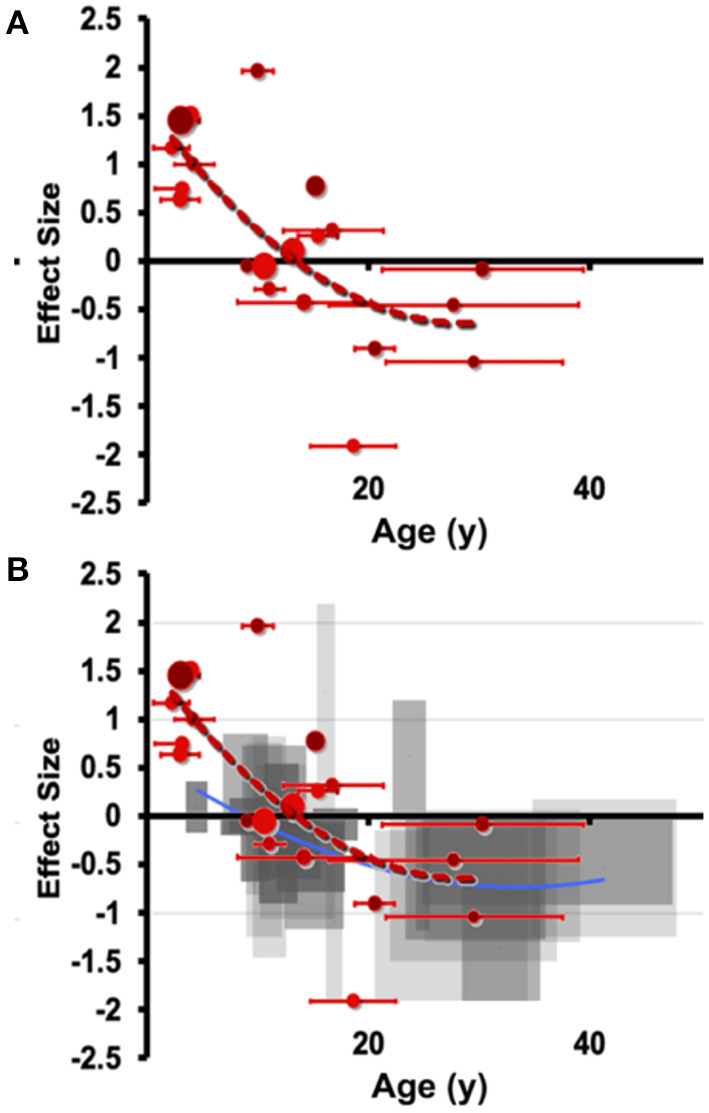
Meta-analytic evidence for a transdiagnostic allostatic curve. Manual hand-tracing studies of **(A)** amygdala volume differences in individuals with autism (ASD) compared to those with autism. Effect size (hedges g) is shown on the vertical axis, with age shown on the horizontal axis (error bars indicate SD of age). Effect sizes are small volume corrected or corrected with total brain volume or intracranial volume. Of note, these samples meet the stricter DSM-IV represented by the smallest reported subgrouping by age. **(B)** Overlaying with the less steep curve of right amygdala from ASD shows a highly similar pattern of shrinkage that is at least as early as the onset seen in ELA.

This pattern of early overgrowth is mirrored in individuals suffering their first episode of psychosis or affective psychosis (combined bipolar and unipolar depression), suggesting a common amygdala response in disorders of sustained distress (Velakoulis et al., 2006). Breaking this down further, Suor et al. (2020) recently showed that early overgrowth was evident in a cross-section of individuals with GAD, social anxiety (or both) and found that a component of social threat was associated with amygdala enlargement. We believe this to be a quantifiable transdiagnostic response to sustained evaluation of social threat, but capturing sizable cohorts suffering their first mood episode before treatment impedes our knowledge of the early stages of the allostatic response. Similarly, emotional overload during social situations is a hallmark of autism spectrum disorders (ASD), which have extensive characterization of degree of social impairment and, conveniently for scientific study, amygdala hyperactivation is expected to start at birth. In ASD, volumetric overgrowths have been reported early in development, but smaller volumes have been noted later in life (Nacewicz et al., 2006; Schumann and Amaral, 2006; Mosconi et al., 2009; Kim et al., 2010) and the rate or degree of early life overgrowth or later life shrinkage is associated with quantitative social impairments. Connected to these ideas, we show in Figure 4 an aggregration of studies in individuals with autism, as well as ELA-exposed samples. We believe the clear pattern of overgrowth and shrinkage in ASD lays the groundwork for a dose-dependent acceleration of this pattern in ELA, consistent with the studies described above showing that adversity with psychopathology or more severe stress from adversity is frequently associated with smaller volumes even on a background of ELA-induced enlargement.

Returning to the case of high ELA exposure, early life volumetric expansion of the amygdala is likely coupled with higher functional reactivity and excitatory tone. Amygdala cells may be able to maintain a moderately high-load state, and individuals with this neurobiological phenotype may display higher levels of depression, anxiety, or other affective illnesses. In some, symptoms may not always reach clinical diagnostic thresholds, or the individuals may be less impaired, albeit with limited capacity to absorb another traumatic insult. In the case of particularly extreme stress exposure, occurring for longer durations of time, initial overgrowth and high metabolic strain may give way to subsequent volumetric shrinkage. In a way, the amygdala may reach a breaking point of over-excitation that leads to smaller structural volumes, while higher functional reactivity and excitatory tone are still present. This fits with the smaller volumes typically noted in adult samples exposed to stress, echoing rodent hippocampal models predicting that *high ratios of inhibitory neurons are metabolically more costly* if unable to reduce high firing rates of excitatory neurons (Wang et al., 2020). As such, we predict that individuals with the largest volumes in early childhood and smallest volumes after the first decade of life will manifest highest levels of symptomatology, likely presenting with one or more full-blown clinical diagnoses.

If amygdala volume after ELA *does* fit the allostatic model, what does this tell us about mechanisms of human psychopathology and treatment potential? What is lost when an the amygdala shrinks but maintains a pathological hyperexcited state? Our understanding of allostatic load grows out of a literature on “learned helplessness,” essentially giving up in the face of a challenge, a core construct in stress-induced depression thought to bridge animal studies and major depressive disorder induced by overwhelming stress (Maier and Seligman, 1976). The key manipulation inducing learned helplessness was a systematic series of alternating mild stressors each day, with the key feature being unpredictability of the next day's stress [as reviewed by Maier and Watkins (2005)]. Sapolsky et al. had identified “damage” to hippocampal neurons (atrophy and loss of apical dendrites) in a case series of vervet monkeys that appeared to die of health consequences of social stress (Uno et al., 1989), but reproducing these with a reliable laboratory model of behavioral stress was *only* achieved using repeated immobility stress (also called “*restraint stress*”; Watanabe et al., 1992). Head to head comparisons with repeated unpredictable stressors showed immobility stress to cause greater dendritic loss in these cells, despite one of the unpredictable stresses on the commonly-used protocol being a single episode of immobility stress (Nibuya et al., 1999). McEwen's group showed antidepressant treatment could reverse hippocampal changes and related spatial learning deficits (Conrad et al., 1996), but, more importantly, a sensitization to fear learning was untouched by the treatment. These investigators concluded “*the results indicate a powerful effect of repeated restraint stress on another brain region, possibly the amygdala, which overrides any influence of the hippocampus*” (Conrad et al., 1999). Vyas et al. built on the sensitized fear conditioning after stress and discovered increased dendritic length, branching, and spines (spines typically represent high fidelity excitatory synapses) throughout the amygdala (Vyas et al., 2002, 2003). These researchers showed immobility stress caused more than double the amygdala remodeling than did unpredictable stress, and again in behavioral testing only the immobility stress increased anxiety-like behavior (Vyas et al., 2002). This research group followed this up with behavioral analysis of these two conditions (Vyas et al., 2004) and showed that 10 days of immobilization stress (2 h/a day) leads rodents to reach a ceiling in anxiety-like behavior while 10 days of unpredictable stress looks just like control. Besides showing an opposite stress-induced remodeling than hippocampal neurons, amygdala remodeling did not reverse 3 weeks after recovery from stress (Vyas et al., 2004). Joining forces with McEwen, Mitra et al. (Mitra et al., 2005) went on to show that even a single 2-h episode of immobility stress induced a delayed expansion in amygdala dendritic spines that paralleled a delayed development of generalized fear. The above findings converge on increased dendritic spines and/or branching on amygdala neurons as a candidate physical mechanism for the generalization of fear itself, which we discuss in more detail below.

However, to highlight the paradigm shift: the expectation up to this point was that repeated *unpredictable* stress induced the strongest behavioral change because of the inability to prepare oneself for what comes next, but unpredictability proved inferior to *inescapability*. Looking across these preclinical models, we would therefore predict that the degree of perceived “*entrapment*” will be a better predictor of amygdala enlargement in individuals exposed to ELA. A systematic review by Taylor et al. (2011) gathers extant evidence from behavioral studies and human studies of depression, psychosis, caregiver burden, chronic pain, anxiety, and traumatic stress that converges on a construct of perceived inescapability that defeat/helplessness contributes to and social support buffers against. Circling back to the “lived experiences” of ELA, when there is not literal entrapment, overwhelming health problems, legal problems, or financial issues may be emblematic of daily life and a form of figurative entrapment. This has potential major implications for poor mental health. Surveying clinical work on self-injurious behavior, elements of figurative entrapment played a role in 25–30% of suicides from 2003 to 2008 (Logan, 2011). Pivoting back to neurobiology, *dendritic expansion may be a compensatory effort to map environmental features to detect safety or avenues of escape*, but in these cases of entrapment, no safety can be achieved so the amygdala churns away, reaching a terminal toxic-metabolic shrinkage in the first decades of life.

A futile cycle attempting and failing to map unattainable safety could explain increased amygdala volume and activity after stress, while other limbic regions (hippocampus, PFC) demonstrate atrophy. We believe research in rodents and non-human primates suggest a testable model about the factors driving this enlargement. As McEwen detailed in multiple reports about allostatic load, the body increases certain functions to meet the demands of the stress, but long-term adaptation is costlier than true homeostasis. Connected to this, Ghosh and Chattarji (2015) examined recruitment of amygdala neurons specifically in the lateral nucleus during fear conditioning, and discovered not only that neurons tuned to a conditioned sound increased their activity after pairing with an aversive stimulus (mild shock), but also that neurons tuned to other sounds broadened their tuning to now respond to the conditioned stimulus. In other words, fear conditioning literally recruits a broader neural network in the amygdala, shifting the balance toward regional excitation. Importantly, a stronger aversive stimulus caused 30% of neurons tested to broadly generalize and respond to nearly any sound, a finding that could be recapitulated by artificially increasing neuronal excitability in the amygdala. A month later, Resnik and Paz (2015) published findings from electrical recordings throughout the three nuclei of the basolateral complex (basomedial, basolateral, and lateral, Figure 2) of macaques, and demonstrated that fear conditioning in primates follows the same pattern of not only strengthening neuronal signatures of environmental cues (sounds) present during an aversive stimulus (in this case a strongly aversive odor) and again broadening of the tuning of neurons not previously responding to sounds in the range of the conditioned stimulus. Just as in the rodent, *the degree of neuronal generalization matched the degree of behavioral fear generalization*. In short, primate and rodent amygdala expand the neural signatures not just of cues indicating danger but recruit more neurons to map the parameter space of similar cues. We believe this is part of a natural mechanism to find the bounds of danger and identify related signals of safety. In support of this, Amir et al. (2015) demonstrate that ~70% of primary neurons and most interneurons in the rodent basolateral nucleus show a graded decrease in firing as the animals leave the safety of their nest and face a robotic predator, with 23% showing an opposite firing rate proportional to danger (possibly the analog of the minority of neurons that strengthened or generalized to cues). Therefore, a subset of amygdala neurons representing danger or generalized fear can drive recruitment of a broader population of amygdala neurons that map relative degrees of known safety.

It is as yet unclear if there is an equivalent fear generalization in primates proportional to the intensity of an aversive stimulus, but this is likely the mechanism by which individuals suffering ELA proportionally over-activate their amygdalae. We predict that this metabolically costly effort to map the environmental space surrounding an intense aversive event represents a key function of the basolateral amygdala as defining the boundaries of danger cues so that safety can be achieved. This is in line with imaging findings and neural network modeling that suggest greatest recruitment in situations of uncertain danger (Kim et al., 2003; Herry et al., 2007). But what if danger is inescapable in all conditions? If no associations with safety are found, and a stimulus is sufficiently intense, perpetual generalized fear responses may ultimately lead to overload and burnout of the “safety mapping” neurons of the amygdala as it endlessly pursues a spatiotemporal boundary to the threat.

## Connecting Amygdala Neurobiology to Developmental Mal/Adaptation After ELA

If amygdala allostatic adaptations do indeed map to attempts to contextualize cues related to threat and vigilance, we believe there are clear developmental translations and connections between this neurobiological phenotype and the aberrant psychosocial and behavioral processes commonly seen after adversity [for a comprehensive review, see Cicchetti (2016)]. These include: disruptions with attachment, emotion processing, and social bonding. While allostatic load models have permeated aspects of developmental psychology, it will be important to increase crosstalk between these areas and to, as Cicchetti noted, “examine the prior sequences of adaptation or maladaptation in development that have contributed to a given outcome.”

Related to attachment, forming a secure early bond provides an individual a base from which to explore and forge new experiences. At the least, this “stable base” literally provides a zone around a parent where “escape” through parental intervention can overcome any threat. Interestingly, individuals exposed to adversity often develop insecure attachment styles [see Cyr et al. (2010), for a meta-analysis], eliminating or at least destabilizing this zone of safety and leading to beliefs of others as unavailable or untrustworthy. This is strongly seen for individuals who have suffered maltreatment, but also for other types of ELA, including low household income, having a parent with a substance use issue, and lower maternal education. These changes could have profound implications for behavioral development, especially if individuals exposed to ELA believe that their caregivers are not safe enough supports for them to explore an environment. In the most extreme cases, infants with disorganized attachments will actually show freezing behavior toward caregivers. This strikingly parallels over-generalization in preclinical fear-conditioning work (Mahan and Ressler, 2012). As these individuals continue to develop, this “unsafe” representation may get expanded out to other individuals in their environments. Thinking about the usefulness of this expanded representation, if one is not sure of who signals safety and security (and what behavior is appropriate to execute in a context), it could be potentially “more adaptive” not to enact any behavior at all. Thinking about attachment and ELA, a number of interesting research findings may relate to aberrant contextual processing. For example, physically abused infants display higher rates of fearfulness, anger, and sadness during parent-infant interactions, compared to age-matched non-maltreated peers (Cicchetti and Ng, 2014). In contrast, neglected infants display blunted ranges of emotional expression, often with an increased duration of negative affect compared to non-maltreated infants. While clearly phenomenologically different, the aberrant processing of contextual safety could potentially explain each of these patterns. Abused infants may be over-contextualizing the negative affect that they experience with their mothers, while neglected infants may be uncertain how and under what circumstances they should be expressing positive emotions.

Moving forward in development and turning to emotion processing, aberrant understanding of contextual cues may connect to the alterations in threat sensitivity and hyper-vigilance commonly reported after ELA. As an illustration, a good deal of recent research, most notably by Pollak et al. (e.g., Pollak et al., 2000; Pollak and Sinha, 2002; Pollak and Tolley-Schell, 2003) finds an increased sensitivity to anger-related cues after abuse: with maltreated children perceiving angry faces as more salient relative to other emotions, display broader perceptual category boundaries for detecting anger, and require less visual information to perceive angry facial expressions. Interestingly, there is suggestive evidence that children exposed to poverty display similar (though subtler) biases toward threat. Indeed, children living in poverty tend to carefully monitor their environment for danger and maintain a low threshold for judging situations as threatening (Chen and Matthews, 2003; Chen and Paterson, 2006). Impoverished youth often exhibit larger cardiovascular responses than higher SES youth when confronted with ambiguous stimuli (Chen et al., 2004). These threat-bias results clearly represent differences in emotion processing but could also be seen as contextual over-generalization or lack of safety learning. Children exposed to adversity may be sensitized to threat, and then they believe threat (and fear) is the common and dominant (and potentially “default”) emotion. In keeping with classic social-information processing work by Dodge et al. (1990), Dodge (1993), and Crick and Dodge (1994), if children exposed to ELA over-contextualize threat and perceive the world to be a hostile and unsafe place, they may respond with high-levels of aggression and potentially retaliatory violence (Connell and Goodman, 2002; Evans et al., 2008; Wilson et al., 2009; Jaffee, 2017; Peverill et al., 2020). The over-representation of subtle signs of threat are likely represented in the synapses and dendritic branching of the amygdala.

Connected to both attachment and emotion processing, those exposed to ELA often have problems with peer relations across multiple stages of development. Interestingly, different forms of ELA (e.g., maltreatment) are related to either withdrawal from or overt aggression toward peers. While many may consider these divergent phenotypes, both may be appropriate (and learned) responses to environmental entrapment and an inability to map safety signals. Youth either fight, attempting to escape perceived entrapment; or eventually surrender under the belief that breaking free is not possible. Furthermore, ELA-exposed youth often make errors in encoding social cues, exhibit biases toward attributing hostile intent, generate more aggressive responses, and positively evaluate aggression as an appropriate response (Teisl and Cicchetti, 2008). Over time, this may lead youth, especially who have suffered ELA and evince amygdala volumetric shrinkage, to select and structure later social interactions to recreate and *validate familiar relationship patterns as a means of reducing amygdala signals of uncertainty*. If the amygdala molecular machinery is also “burned out”, it may be challenging to differentiate safety vs. threat. Indeed, adults exposed to ELA report greater interpersonal sensitivity, paranoia, and hostility (e.g., Liem and Boudewyn, 1999). Such patterns are most consistently noted in previously maltreated samples, but similar results have been reported in cumulative ELA (the “ACEs”) with cumulative childhood trauma exposure by 16 years of age relating to negative social/peer outcomes (e.g., poorer quality of relationship with spouse/significant other and friends; Copeland et al., 2018). Additional research indicates adults that suffer early social neglect are less likely to be married than their non-neglected peers (e.g., Tieman et al., 2005), and report fewer social supports and close confidants (Weiner and Kupermintz, 2001; Sigal et al., 2003). This suggests that overrepresentation of threat may come at the cost of learned social safety and affiliation mappings in networks connecting through the amygdala.

## Environmental Moderation of Safety Learning: Influence of Caregivers

While excitation-inhibition imbalances in the amygdala may be a consequence of ELA, the potential neural embedding of inescapability is not without counterweights. Breakthroughs in the last two decades have identified specific “*extinction cells*” in basolateral amygdala that receive inputs from portions of the prefrontal cortex (Herry et al., 2008; Likhtik et al., 2008; Strobel et al., 2015); in preclinical models, this includes cells in infralimbic (IL) PFC, with signals from this portion of PFC driving the Basomedial amygdala nucleus (sometimes termed “accessory basal nucleus”) to inhibit fear by cues and contexts known to confer safety (Adhikari et al., 2015; Bloodgood et al., 2018). In primates, the evolutionary descendent of infralimbic PFC, subgenual cingulate or Brodmann Area 25 (BA25), was recently found to have a similarly heavy projection to the basomedial nucleus in macaques (Kim et al., 2018). Direct chemical overactivation broadly of BA25 in primates *increases* sympathetic tone, fear sensitivity and amygdala activation (Alexander et al., 2020), attesting to the ability of BA25 to also signal threat (likely via the paraventricular thalamus) and that broad overactivation favors fear over safety. Building on our model that the basolateral amygdala is recruited to build a finer mapping of the gradation from threat to safety, the BA25 signal may dampen fear-responding to known threats if previously learned escape mechanisms (e.g., stay away from the house when parent is intoxicated) are available. Conveniently, co-activation of infralimbic PFC and basolateral amygdala in rodents inhibits the ventral tegmental area (VTA), reducing the key dopamine signal that “unlocks” the basolateral complex (Marowsky et al., 2005; Patton et al., 2013).

**Figure 5:**
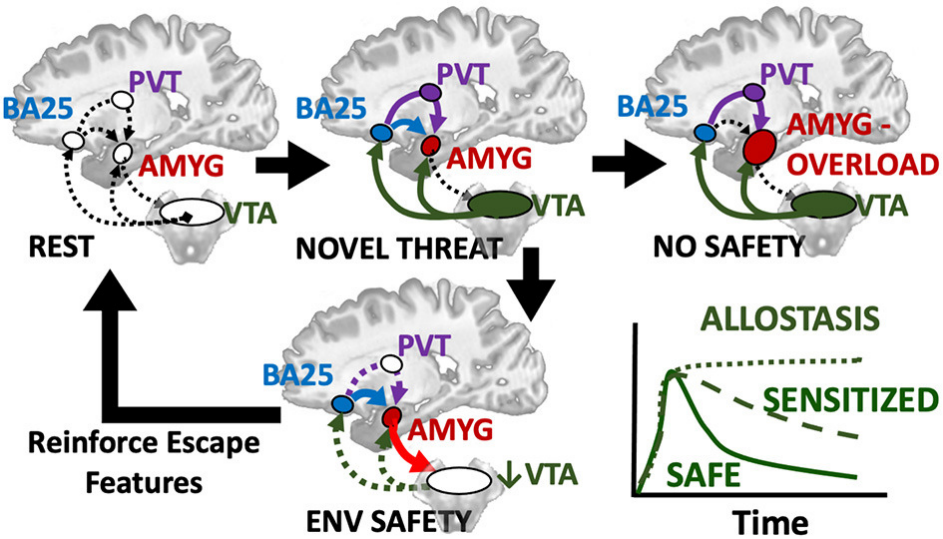
Hypothesized loop by which a novel threat activates alerting/approach related ventral tegmental area (VTA) which provides dopaminergic innervation to prefrontal regions including the subgenual BA25 sends the resultant evaluation of situational safety through the uncinate fasciculus (blue) to basal and lateral amygdala nuclei and simultaneous threat signals through the paraventricular thalamus (PVT, purple) to the centromedial amygdala effectors. Co-activation of BA25 and amygdala safety circuits deactivates the VTA and cues encoding safety or escape are retained and reinforced as reward. If no safety is achievable, chronic activation of BA25 and amygdala including dopamine signals from the VTA lead to fear generalization or sensitization and favor the PVT relay and latent fears. A transient response or deactivation of dopamine appears to be a key manifestation of safety.

This raises the possibility of a protective circuit (Figure 5) by which: (1) a novel stimulus triggers dopamine release from the VTA, leading to (2) activation of prefrontal and limbic regions including disinhibiting basolateral amygdala to alter the scope of fear, (3) safety and threat information is synthesized in subgenual BA25 and transmitted to primary cells in basolateral amygdala, which (4) may conclude the threat is manageable and deactivate the entire loop by turning off the VTA. Alternatively, sustained responses favor centromedial output nuclei, the site of greatest dopaminergic innervation in the human amygdala (García-Amado and Prensa, 2013), likely signaling innate and previously learned fears that flow through a BA25->paraventricular thalamus->centromedial amygdala circuit (Do-Monte et al., 2015; Penzo et al., 2015).

With this circuit in mind, we consider the groundbreaking work of Regina Sullivan, who showed in a rat model that there is a critical period during which associating a cue with a natural threat (e.g., fox odor) can paradoxically produce an *appetitive response* when conditioned in the presence of mother (Moriceau and Sullivan, 2006). It is unknown, as of yet, if there is an equivalent critical period in human development during which facing threatening situations in the safety of a parent causes reminders of the experience to induce positive and even antidepressant-like responses later in life (Rincón-Cortés et al., 2015). Sullivan's group traced this effect to a temporary drop in dopamine in the basolateral complex when the mother was present (Barr et al., 2009). More recently, her group found that developmental maturation of the infralimbic cortex->amygdala pathway supplants this effect (Robinson-Drummer et al., 2019), such that “social buffering” by mother beyond the critical period induces a negative correlation between amygdala and VTA activation, as measured with 2-deoxyglucose metabolic mapping (Opendak et al., 2019). In contrast, maternal maltreatment induced by resource scarcity closes the window such that maternal presence no longer deactivates amygdala within the critical period and at later ages maternal presence (perhaps appropriately) no longer deactivates the amygdala, nor does it produce the negative coupling of basolateral amygdala and VTA that would indicate convergent safety signals from BA25 and basomedial amygdala. While we are far from understanding all the components of this response, it builds on the model that the BA25-basolateral amygdala pathway likely carries signals of relative safety from a threat in the environment, consistent with human studies of structural connectivity (Tromp et al., 2019). Another recent human study suggests the major influence of parental warmth on subsequent brain function and psychopathology in preadolescence, when this system comes online, is most evident in subgenual cingulate (BA25) activation and functional connectivity with amygdala (Butterfield et al., 2020). The neural substrate of positive parental and environmental influences that prevent psychopathology is likely BA25->basomedial amygdala circuit deactivation of VTA.

We can take this model one step further and consider how social buffering could lower the excitation:inhibition ratio of the amygdala so as not to rush experience-dependent plasticity during development. Zhang et al. (2020) mapped representations of aversive stimuli to a subtype of inhibitory cell in basolateral amygdala (expressing RSPO2) and safety signaling to a subtype expressing Dopamine Associated neuronal PhosphoProtein (DARPP-32; as known as Ppp1r1b). They then showed that direct reactivation in the absence of threat of the DARPP-32+ neurons that comprise the extinction (safety) memory trace produced a strong reward response in a contextual conditioning test. This raises the possibility that healthy amygdala development involves depositing layers of neurons, storing rewarding solutions to previously survived experiences and exposures that inhibit broad amygdala activation and fear generalization. It is not yet known whether these neurons are direct recipients of BA25 innervation or whether they differentially express dopamine receptors, but we speculate that social buffering in humans likely requires activation of the loop described above producing a pulse of dopamine and then a drop in basolateral amygdala dopamine and that this ultimately strengthens the DARPP-32 neurons in the basolateral complex.

Feedforward safety signals likely reduce the average excitation:inhibition ratio of the amygdala, permitting a slower maturation throughout adolescence and possibly more DARPP-32 cells that could activate positive affects in safe contexts. If an environment offers few to no safety signals, this maturation is accelerated according to the allostatic load model, progressing more rapidly to dendritic outgrowth, higher excitation:inhibition and an early allostatic peak that likely compresses stress buffering periods. It is unknown, however if the lack of any parental safety signals during this critical period, most exemplified by institutional neglect, leads to amygdala shrinkage in the absence of additional adversity. This is particularly challenging given the high incidence of further adversity in foster care and may be better studied in children with diffuse attachment disorder. We speculate that the lack of safety learning during critical periods leads to limited psychological and emotion regulation resources, represented by the safety learning possibilities wired through the DARPP-32+ cells, to overcome challenges. Lack of social safety learning likely leads to inability to detect and avoid dangerous environments and individuals, ultimately leading to similar outcomes.

Fortunately, future treatments may be able to mimic the temporal dynamics of dopamine signaling and repair or renew DARPP-32 cells in the basolateral complex, re-opening a window of plasticity for social safety learning. Consistent with this model, a single dose of the dopamine precursor L-DOPA/carbidopa facilitates fear extinction in rodents and humans through enhanced negative connectivity between ventromedial prefrontal regions and amygdala (Haaker et al., 2013). More recent work by Cisler et al. (2020) showed that L-DOPA/carbidopa enhances post-training reactivation of amygdala during memory consolidation, leading to enhanced extinction of fear in adults with PTSD. It is tempting to presume that this enhanced amygdala activation includes a preponderance of DARPP-32+ safety cells, but more research in this area is needed. It will be critical to integrate these findings into emerging theories about the neurobiological impacts of ELA, as well as models examining connections between ELA and psychopathology.

## Further Consideration of Ela, Amygdala Neurobiology, and Clinical Practice

As noted earlier, we took a more broad and inclusive definition of ELA here, but additional work is clearly needed to richly characterize and define stressful early life experiences. This work will come in many forms and will need to consider developmental context, “true” lived experiences, subjective perceptions, and the temporal dynamics of adversity exposure. Further cataloging of positive events and success overcoming challenges despite adversity will enrich our understanding of stress buffering and safety learning. Indeed, all of these factors will likely influence neurodevelopment and may impact trajectories of amygdala neurobiology.

First, and to be critical, many studies (e.g., Hair et al., 2015; Hanson et al., 2019) actually focus on developmental exposures, or the adverse contexts that youth develop in. This is in contrast to true experiences that a child *actually* encounters. To borrow an illustrative example from (McLaughlin et al., 2020), two children may be exposed to similar negative life circumstances (e.g., parental drug abuse), but experience very different things (i.e., parental hostility, vs. caregivers receiving drug treatment). This is very much the case for exposure to poverty and economic marginalization. Numerous studies have shown that poverty, an adverse exposure, is associated with a host of stressful experiences including: neighborhood violence, housing instability, and issues with household structure and organization (Evans and English, 2002). Illustrating this idea for child neglect, we think about the context of institutional rearing vs. parental neglect. Youth in institutional settings may actually have support from staff or peers within these congregate care settings (e.g., McCall et al., 2019) while those living with a neglectful parent may rarely feel safety and experience greater perceived entrapment. Clear measures of specific negative experiences, during adverse exposures, will surely aid in understanding the types and “dosing” of negative experiences likely to influence brain and behavior. In addition to this distinction, the field should expand assessments of the subjective perceptions of ELAs. Strong recent work by Danese and Widom (2020) underscores that participants' perceptions of their experience may be the most predictive of behavioral challenges. In a unique cohort of individuals followed since childhood with court-documented evidence of maltreatment and subjective reports of childhood maltreatment histories, these investigators found subjective reports of childhood maltreatment were more robust predictors of psychopathology, regardless of whether objective (court) records substantiated maltreatment. Thinking more about exposures, experiences, and subjective perceptions may aid in clarifying inconsistent neurobiological findings.

Related to subjective perceptions, as well as the dichotomy of exposure vs. experience, dimensional models begin to overcome many of the limitations in past studies, but there is more work to do in this space especially related to developmental timing. For example, McLaughlin et al. (2014a), as well as Belsky et al. (2012) and Ellis et al. (2017), articulate potential dimensions of experience that may influence development (e.g., Deprivation vs. Threat; Harshness vs. Unpredictability); however, it is unclear if differences in developmental competencies, especially early in life, may cause the blurring of boundaries between ELA dimensions. For example, children exposed to early neglect while living in institutional care would, in theory, represent a “*deprivation*” ELA dimension. However, these children are often very young (<3 years of age) when they are in these settings, and the global neglect they are experiencing may impinge upon attachment processes (represented in basomedial and superficial nuclei, Figure 3). Such experiences, perhaps due to subjective perceptions and processes, would then actually be threatening in nature—the lack of a clear attachment figure would cause heightened vigilance to environmental dangers. This actually fits well with results from Tottenham's group that has found post-institutionalized children who suffered early social neglect have alterations in the amygdala, both structurally and functionally (e.g., Cohen et al., 2013; VanTieghem et al., 2021) and more recently found amygdala volume predicted later stress hormone responses (VanTieghem et al., 2021). Of important note, we believe compelling distinctions exist in non-human animal models of stress for inescapable vs. unpredictable stressors, but believe it still too early to synthesize these ideas to the exposure vs. experience distinction in humans. We are hopeful that future conceptual work could continue progress in this space.

Furthermore, while research teams often catalog many specific occurrences of stress, many types of common (*day-to-day*) experiences may not rise to the level of a “formal ELA.” For example, there is a litany of research on “expressed emotion” and risk for poor mental health (Butzlaff and Hooley, 1998; Weintraub et al., 2017). Expressed emotion is hostility, criticalness, and excessive involvement of family members toward someone in the family with identified mental health problems (Weintraub et al., 2017). These family relational patterns can represent a psychosocial stressor that interacts with individuals' diatheses, eventually culminating in relapse (Hooley and Gotlib, 2000). Put another way—growing up with a parent suffering from substance use disorder, or who is hostile, may mean unpredictable bursts of anger or threatening behavior; this may instill a chronic stressful alertness, or influence attachment processes, as youth look for any sign of a bad mood or known aggravating factor. Clinically, research has found that hostility and emotional over-involvement slowed progress with interventions such as exposure therapy (Tarrier et al., 1999). However, and connected back to our conceptualization of the amygdala, hostile and emotionally boundaryless contexts may be perceived as psychologically unsafe and, ultimately, an inescapable stressor. It will be important to think about this and related elements of normal and atypical parenting in relation to neurobiology [for thoughtful review in this space, see Farber et al. (2020)].

Forging connections between these elements of adversity, our neurobehavioral conceptualization of the amygdala, and clinical outcomes, high or chronic levels of ELA, or ELA coupled with recent stressors, may eventually embed perceptions or feelings of inescapability and “being trapped.” In many cases, this may take multiple psychosocial forms, from legal and financial problems to chronic pain, to anxiety, depression, and other forms of psychopathology. Amygdala neurobiological changes (e.g., initial dendritic expansion; hyper-excitability) may be compensatory efforts to map environmental features to detect safety in the context of these stressors; however, with high-levels of psychosocial burden and/or limited social support (limited formation of DARPP-32+ escape solutions), no safety can be achieved so the amygdala churns away, reaching a terminal toxic-metabolic shrinkage. These psychosocial perceptions and neurobiological changes may indeed explain many “Deaths of Despair” in populations exposed to ELA (Bohnert and Ilgen, 2019). Declining opportunity or inability to escape deleterious life circumstances cause many to turn to opioids or other drugs to cope. In extreme cases (and exposure to multiple ELAs), this may lead to extreme learned helplessness and suicidality. This broad conjecture fits well with extant data showing elevated drug use and abuse, as well as suicide attempts in many exposed to ELAs (Dube et al., 2001, 2003; Brodsky and Stanley, 2008). For example, work has found that ~30% of suicide attempts among women and 23% of those among men were attributable to having experienced repeated ELAs (e.g., physical abuse, sexual abuse, witnessing domestic violence; Afifi et al., 2008). Clinicians working with individuals with high ELA may be able to interrupt this deleterious cycle by helping clients see potential “escapes” out of multiple/compounding, real (or perceived) psychosocial challenges.

## Thinking About Stigma, Resilience, and “Hidden Talents”

While we believe that the model that we advance here is critical to understanding the sequalae of ELA, it is also important to acknowledge the balancing act that researchers and connected groups are intending to strike in thinking about the impact of ELAs. First, we are mindful of the potential stigma in connecting neurobiology to experiences of trauma, poverty, and other adversities. Without realizing it, one can create what many have termed “*biosocial determinism*” and the unintended, neurobiological rationalization of adversity-related disparities (Pitts-Taylor, 2019). Our aims are, instead, that leveraging ours and connected ideas related to the neurobiology of ELA can not only promote prevention, but also push larger structural changes at institutional levels to reform potential societal factors correlated with ELAs. For example, scientific research on institutionalization, and the child neglect common to many of these congregate care settings, has spurred many policy-driven changes aimed at transforming child protective services and bolstering support for families and communities (Llorente et al., 2003; McCall et al., 2013; Berens and Nelson, 2015). Second, there is the additional connected risk of implicitly disparaging low-income households. Poverty and experiencing economic marginalization do not produce child maltreatment, and abuse occurs across all strata of the socioeconomic spectrum. Similarly, poverty is not synonymous with stress; however, poverty is associated with experiencing greater numbers of stressful life events and other hazards to youth development (Evans and English, 2002). We are mindful that parents and caregivers are often seen as responsible for these elements, while systemic factors contributing to adversity, such as social inequality and racism, routinely receive less attention and focus. We are encouraged by recent perspectives emerging from science and technology studies pushing for the transformation of research on ELA in how it is absorbed and discussed in different social and policy circles (Müller and Kenney, 2020).

Of additional note, neurobiological methods can homogenize people who have suffered ELA and often obscure the variety of strengths present within individuals. There is a growing literature on “*hidden talents*” that underscores many individuals growing up in high-adversity context have intact, or even enhanced, social, cognitive, and affective skills (Ellis et al., 2017, 2021; Frankenhuis et al., 2020). For example, economically marginalized adults often show enhanced procedural learning (i.e., acquiring stimulus–response associations) compared to their higher socioeconomic status peers. Scholars in this area, notably Ellis and Frankenhuis, have underscored that differences in neurobiology or behavior may be useful adaptations in responses to the adverse contexts; these differences may promote adaptive functioning, as one thinks about the different contexts that individuals inhabit. Relatedly, there is a large and well-developed body of research on resilience to ELA (Masten et al., 1999; Southwick et al., 2014), as well as post-traumatic growth (Tedeschi and Calhoun, 2004). This is outside the scope of our review, but at the broadest level, these bodies of research underscore most individuals who suffer ELA do not experience mental or physical health disorders. Negative outcomes, like higher aggression or greater depression, are enhanced, but these are not deterministic links. Many factors (e.g., social support, cognitive functioning) protect youth in the context of severe adversity (for thorough discussion, see the work of Ann Masten, e.g., Masten et al., 2021), We, as a field must be mindful that: (1) many exposed to ELA do not evince significant impairments; (2) there may be positive change that occurs as a result of the struggle with highly challenging life crises (Infurna and Jayawickreme, 2019); and (3) successful outcomes may be achieved through differential trajectories of neural and behavioral functioning. Holding these collective concepts in mind can limit the further marginalization of ELA-exposed populations and will no doubt aid in supporting youth exposed to adversity.

## Specific Future Directions Motivated By Our Conceptual Model

The innovative and interesting conceptual framework put forth here suggests a number of critical future directions in the study of ELA and amygdala neurobiology. These include formal tests of the proposed model, to considered revisions of some preclinical stress manipulations. We elaborate on these below.

First, and related to our model, there is the potential to adapt and deploy magnetic resonance spectroscopy (MRS) to probe markers of amygdala neurobiology, including cell density, membrane phosphocholines, second messenger turnover, and other critical molecular markers. The second author and his colleagues at University of Wisconsin-Madison (e.g., Nacewicz et al., 2012) have been able to measure pooled glutamate and glutamine to probe potential excitotoxicity in typical and atypical samples (e.g., Individuals with Autism). This work involves custom fitting of MRS voxel to amygdala anatomy as we found an extraordinary sensitivity to partial inclusion of different amygdala nuclei. Moving forward, although capturing the average neurochemical concentrations of an entire amygdala is spatially imprecise, the temporal and neurochemical precision offered by emergent functional spectroscopy techniques promises to unravel the exact dynamics of excitatory glutamate, inhibitory GABA and even the acetylcholine (Bell et al., 2019), which is enriched in the basolateral nucleus. Additionally, novel diffusion weighted techniques (NODDI; Zhang et al., 2012) estimate neurite (dendritic) complexity in gray matter with a resolution approaching structural anatomical images, and could longitudinally characterize the normal vs. allostatic peak amygdala volumes. Complementing this, the novel PET tracer UCB-J binds the synaptic marker SV2A as a direct, albeit relative, measure of synapse density (Finnema et al., 2016). Combining these methods, longitudinal increases or decreases in synapses and dendritic trees can be combined with glutamate and GABA measurements to determine the relative outgrowth of inhibitory vs. excitatory synapses, thereby directly tracking development of allostatic load. These tools can help screen for environmental influences and treatments that halt the amygdala overgrowth or at least the toxic shrinkage.

Second, and related to stress manipulations in preclinical work, there are many experimental design changes that could shed light on the full scope of neurobiological consequences of ELA. To be blunt, few, if any, rodent models are developmentally sensitive (cf. work by Regina Sullivan et al.). The seminal studies by Vyas et al. motivated a host of human studies on ELA; however, this work was completed in young adult rodents. Additional work that is often less highlighted, by Rosenkranz et al. completed similar stress manipulations and found many critical differences (e.g., number of spontaneously firing neurons vs. firing rates) in animals exposed to stress as juveniles vs. in adulthood. Little work, in our opinion, goes far enough to truly understand the true developmental impact of ELA. In line with recommendations from Callaghan et al. (2019), it is and will be critical to consider the developmental ecology and “goals” of an organism (e.g., attachment; independence) in thinking about neurodevelopment. Such ideas also connect to a translational challenge in most preclinical studies—nearly all of these paradigms do not adequately capture the transactional nature of stress (Lazarus and Folkman, 1987). To be concrete, in humans, ELA (e.g., the multiple stressors associated with poverty; maltreatment) may affect how an individual responds to subsequent life stressors (Monroe and Harkness, 2005; Hammen, 2018).

In particular, we believe it will be important to consider the interaction of ELAs with stressors during childhood and also later in life. ELA may affect how individuals respond to subsequent life stressors and heighten risk for poor mental health (Monroe and Harkness, 2005; Hammen, 2018). Rich support has been found for this “stress sensitization” or “two-hit models,” for example, women with exposure to one or more childhood adversities (e.g., family violence, parental psychopathology) were more likely to become depressed by a lower “dose” of total stress than women without such adversity (Hammen et al., 2000). This is true for depression, anxiety, and PTSD, and found during childhood, adolescence, and adulthood (Dougherty et al., 2004; Kendler et al., 2004; Harkness et al., 2006; Espejo et al., 2007; McLaughlin et al., 2010). Framed in terms of our safety-mapping model: avoiding a known threat (guided by BA25-basomedial amygdala signals), such as avoiding a parent or home at the slightest sign of a bad mood, reduces the proportion of one's life environment in which an individual feels safe. A subsequent adverse event in another environment thought to be “safe,” e.g., escaping an abusive home and ending up in a romantic partnership that involves further abuse, quickly exhausts the system and hastens the allostatic changes. We also note that a single act of random unprovoked violence can sometimes prove more difficult to treat than repeated predictable abuse from a known individual, again likely representing the challenge of finding anything other than temporal signs of safety. In this way, ELA may sensitize individuals to later challenges whereby mild to moderate stressors complete a mapping of inescapability, intensifying fear generalization by glucocorticoid-induced amygdala remodeling. Put more clearly—the occurrence of ELA may fundamentally change how an organism moves through and approaches the world. There may be changes in coping, self-concept, and other complex psychosocial processes that then interact with later events. Limited neurobiological work has examined such ideas (cf., Hanson et al., 2015a, 2018, 2019), but it would be a fruitful target for future investigations.

Third, a hopeful discovery by Sara et al. two decades ago (Przybyslawski et al., 1999) suggested that conditioned fear becomes labile when reactivated and its reconsolidation can be disrupted such that the stored fear was no longer susceptible to stress-induced reinstatement. Put simply, we can erase fear conditioning by combining reactivation and pharmacotherapy. While much had to be learned in the process, which was traced to synapse-specific protein synthesis triggered by calcium influx primarily from NMDA receptors (Nader et al., 2000; Debiec et al., 2006), it ultimately yielded promising candidates for medication-assisted exposure therapy. This emerging work has identified NMDA-antagonistic agents (D-cycloserine, ketamine; Guastella et al., 2008; Kalisch et al., 2009; Otto et al., 2010; Das et al., 2019), a mitochondrial enhancer (methylene blue; Telch et al., 2014; Zoellner et al., 2017) and the anti-adrenergic propranolol as having potential to erase the fight-or-flight component of fear learning (Kindt et al., 2009; Soeter and Kindt, 2015; Brunet et al., 2018). Several caveats were discovered including: (1) the memories must be reactivated or the treatment adds nothing (2) the medication exposure must end well, with relative safety achieved by the end or treatment could worsen the fear and (3) conveniently, the medication need not be administered prior to the exposure but in a 4–6 h window of neuroplasticity after treatment (4) in the case of propranolol a much higher dose is required (40 mg acute dose) than would typically be prescribed for situational phobia. Armed with these tools, we may be able to eliminate the biological fear response and accelerate treatment (Brunet et al., 2018), but interestingly subjective fear can lag the biology significantly (Rothbaum et al., 2014; Soeter and Kindt, 2015). This opens an unprecedented avenue to understand the separate components of biological fear generalization and subjective impressions of entrapment. Further, as noted above, other agents such as L-DOPA hold the promise of enhancing the extinction memory rather than the disrupting the fear memory itself. Given that activation of extinction neurons in the absence of fear neurons is rewarding in rodents, head-to-head comparisons of different medication-assisted therapies could disentangle the relative contributions of safety signals and fear signals in the allostatic load model. More importantly, our limited mental health resources may be able to increase efficiency for treating the enormous toll of ELA and adult mental illness currently inflicted by the COVID-19 pandemic.

Finally, it will be important to investigate more deeply potential “tipping points” in human ELA exposed samples and whether cascades related to ELA may be pernicious and excitotoxic. Adversity may potentially start at (and continue into) multiple, different stages of development. In addition, there may be psychosocial consequences of ELA that may increase the likelihood of volumetric shrinkage in exposed individuals. Such a developmental incorporation may have important insights for the prediction, prevention, and treatment of negative outcomes related to ELA. ELA may cause psychosocial (or neurobiological) alterations at one time point, but individuals who have suffered these adversities keep engaging in, as well as creating, different experiences. Outcomes at later time points may be related to this initial adaptation, but also could be due to the interaction of early changes and current situational experiences. Clinically, those working with adversity-exposed populations often know that previous (negative) adaptations in their clients may create later residue that individuals who have suffered bring to the different situations that subsequently greet them. Connected to the ideas we advance above, future work will need to consider not just age and duration of a stressor, but also the “escapability” or proportion of contexts affected (e.g., daycare, home, and a relative's house). Thoughtful execution of these multiple ideas, across preclinical and human studies, could significantly advance our understanding of the neurobiological sequelae of ELA, the mediating connections between ELA and psychopathology, and more basic science questions such as nature vs. nurture.

## Concluding Remarks

Here, we put forward an integrated model of amygdala neurodevelopment to think about inconsistencies in research on ELA, as well as the behavioral consequences of adversity. We must all continue to dissect heterogeneity, think about theoretical integrations of stress neurobiology and developmental psychology, and clarify complex relationships between ELA and related long-term mental health challenges. We are excited to pursue many of the future directions we proposed here and would be excited about improvements in preclinical and human studies focused on early stress exposure. Continued progress in these spaces, potentially guided by the theoretical model laid out here could be particularly important for predicting, preventing, and treating the consequences of ELA.

## Data Availability Statement

The datasets presented in this study can be found in online repositories. The names of the repository/repositories and accession number(s) can be found at: https://github.com/jlhanson5/Hanson_Nacewicz_Frontiers_Amygdala_Review_Data.

## Author's Note

The authors owe a great debt to the late Dr. Bruce McEwen, whose careful measurement of the effects of sustained stress and theoretical extrapolation far beyond the timescale of typical research studies led to the model of Allostasis. His bridge from basic science studies of rodent neurons and synapses to human neuroimaging and behavior inspired us as neuroscientists. As this model has developed, it has become a fantastic tool for patient education in psychiatric practice (of BN) as a tangible mechanism by which fears expand or improve with treatment.

## Author Contributions

Both authors contributed equally to the outlining, drafting, and writing of the manuscript.

## Conflict of Interest

The authors declare that the research was conducted in the absence of any commercial or financial relationships that could be construed as a potential conflict of interest.

## References

[B1] Adhikari A. Lerner T. N. Finkelstein J. Pak S. Jennings J. H. Davidson T. J.. (2015). Basomedial amygdala mediates top-down control of anxiety and fear. Nature 527, 179–185. 10.1038/nature15698 26536109 PMC4780260

[B2] Afifi T. O. Enns M. W. Cox B. J. Asmundson G. J. Stein M. B. Sareen J. (2008). Population attributable fractions of psychiatric disorders and suicide ideation and attempts associated with adverse childhood experiences. Am. J. Public Health 98, 946–952. 10.2105/AJPH.2007.120253 18381992 PMC2374808

[B3] Alexander L. Wood C. M. Gaskin P. L. R. Sawiak S. J. Fryer T. D. Hong Y. T.. (2020). Over-activation of primate subgenual cingulate cortex enhances the cardiovascular, behavioral and neural responses to threat. Nat. Commun. 11:5386. 10.1038/s41467-020-19167-0 33106488 PMC7588412

[B4] Almeida D. M. (2005). Resilience and vulnerability to daily stressors assessed *via* diary methods. Curr. Dir. Psychol. Sci. 14, 64–68. 10.1111/j.0963-7214.2005.00336.x

[B5] Amaral D. G. (2002). The primate amygdala and the neurobiology of social behavior: implications for understanding social anxiety. Biol. Psychiatry 51, 11–17. 10.1016/S0006-3223(01)01307-5 11801227

[B6] Ambroggi F. Ishikawa A. Fields H. L. Nicola S. M. (2008). Basolateral amygdala neurons facilitate reward-seeking behavior by exciting nucleus accumbens neurons. Neuron 59, 648–661. 10.1016/j.neuron.2008.07.004 18760700 PMC2603341

[B7] Amir A. Lee S.-C. Headley D. B. Herzallah M. M. Pare D. (2015). Amygdala signaling during foraging in a hazardous environment. J. Neurosci. 35, 12994–13005. 10.1523/JNEUROSCI.0407-15.2015 26400931 PMC4579372

[B8] Appel A. E. Holden G. W. (1998). The co-occurrence of spouse and physical child abuse: a review and appraisal. J. Fam. Psychol. 12:578. 10.1037/0893-3200.12.4.578

[B9] Avino T. A. Barger N. Vargas M. V. Carlson E. L. Amaral D. G. Bauman M. D.. (2018). Neuron numbers increase in the human amygdala from birth to adulthood, but not in autism. Proc. Natl. Acad. Sci. U. S. A. 115, 3710–3715. 10.1073/pnas.1801912115 29559529 PMC5889677

[B10] Babalola K. O. Patenaude B. Aljabar P. Schnabel J. Kennedy D. Crum W.. (2009). An evaluation of four automatic methods of segmenting the subcortical structures in the brain. NeuroImage. 47, 1435–1447. 10.1016/j.neuroimage.2009.05.029 19463960

[B11] Bachevalier J. Alvarado M. C. Malkova L. (1999). Memory and socioemotional behavior in monkeys after hippocampal damage incurred in infancy or in adulthood. Biol. Psychiatry 46, 329–339. 10.1016/S0006-3223(99)00123-7 10435198

[B12] Barr G. A. Moriceau S. Shionoya K. Muzny K. Gao P. Wang S.. (2009). Transitions in infant learning are modulated by dopamine in the amygdala. Nat. Neurosci. 12, 1367–1369. 10.1038/nn.2403 19783994 PMC2783302

[B13] Bell T. Lindner M. Langdon A. Mullins P. G. Christakou A. (2019). Regional striatal cholinergic involvement in human behavioral flexibility. J. Neurosci. 39, 5740–5749. 10.1523/JNEUROSCI.2110-18.2019 31109959 PMC6636079

[B14] Belsky J. Schlomer G. L. Ellis B. J. (2012). Beyond cumulative risk: distinguishing harshness and unpredictability as determinants of parenting and early life history strategy. Dev. Psychol. 48:662. 10.1037/a0024454 21744948

[B15] Berens A. E. Nelson C. A. (2015). The science of early adversity: is there a role for large institutions in the care of vulnerable children? Lancet 386, 388–398. 10.1016/S0140-6736(14)61131-4 25638660 PMC9594997

[B16] Björkenstam E. Vinnerljung B. Hjern A. (2017). Impact of childhood adversities on depression in early adulthood: a longitudinal cohort study of 478,141 individuals in Sweden. J. Affect. Disord. 223, 95–100. 10.1016/j.jad.2017.07.030 28735168

[B17] Bloodgood D. W. Sugam J. A. Holmes A. Kash T. L. (2018). Fear extinction requires infralimbic cortex projections to the basolateral amygdala. Transl. Psychiatry 8, 1–11. 10.1038/s41398-018-0106-x 29507292 PMC5838104

[B18] Bohnert A. S. Ilgen M. A. (2019). Understanding links among opioid use, overdose, and suicide. N. Engl. J. Med. 380, 71–79. 10.1056/NEJMra1802148 30601750

[B19] Bremner J. D. Randall P. Vermetten E. Staib L. Bronen R. A. Mazure C.. (1997). Magnetic resonance imaging-based measurement of hippocampal volume in posttraumatic stress disorder related to childhood physical and sexual abuse—a preliminary report. Biol. Psychiatry 41, 23–32. 10.1016/S0006-3223(96)00162-X 8988792 PMC3229101

[B20] Brodsky B. S. Stanley B. (2008). Adverse childhood experiences and suicidal behavior. Psychiatr. Clin. North Am. 31, 223–235. 10.1016/j.psc.2008.02.002 18439446

[B21] Brunet A. Saumier D. Liu A. Streiner D. L. Tremblay J. Pitman R. K. (2018). Reduction of PTSD symptoms with pre-reactivation propranolol therapy: a randomized controlled trial. Am. J. Psychiatry 175, 427–433. 10.1176/appi.ajp.2017.17050481 29325446

[B22] Buser N. J. Madan C. R. Hanson J. L. (2020). Quantifying numerical and spatial reliability of amygdala and hippocampal subdivisions in freesurfer. bioRxiv 2020.06.12.149203. 10.1101/2020.06.12.149203 PMC10082143 37029203

[B23] Butler O. Yang X.-F. Laube C. Kühn S. Immordino-Yang M. H. (2018). Community violence exposure correlates with smaller gray matter volume and lower IQ in urban adolescents. Hum. Brain Mapp. 39, 2088–2097. 10.1002/hbm.23988 29450935 PMC6866594

[B24] Butterfield R. Silk J. Lee K. H. Siegle G. Dahl R. Forbes E.. (2020). Parents still matter! parental warmth predicts adolescent brain function and anxiety and depressive symptoms two years later. Dev. Psychopathol. 32, 1–14. 10.1017/S0954579419001718 32096757 PMC7483163

[B25] Butzlaff R. L. Hooley J. M. (1998). Expressed emotion and psychiatric relapse: a meta-analysis. Arch. Gen. Psychiatry 55, 547–552. 10.1001/archpsyc.55.6.547 9633674

[B26] Caldwell J. Z. K. Armstrong J. M. Hanson J. L. Sutterer M. J. Stodola D. E. Koenigs M.. (2015). Preschool externalizing behavior predicts gender-specific variation in adolescent neural structure. PLoS ONE 10:e0117453. 10.1371/journal.pone.0117453 25658357 PMC4319931

[B27] Callaghan B. Meyer H. Opendak M. Van Tieghem M. Harmon C. Li A.. (2019). Using a developmental ecology framework to align fear neurobiology across species. Ann. Rev. Clin. Psychol. 15, 345–369. 10.1146/annurev-clinpsy-050718-095727 30786246 PMC7219957

[B28] Carr C. P. Martins C. M. S. Stingel A. M. Lemgruber V. B. Juruena M. F. (2013). The role of early life stress in adult psychiatric disorders: a systematic review according to childhood trauma subtypes. J. Nerv. Ment. Dis. 201, 1007–1020. 10.1097/NMD.0000000000000049 24284634

[B29] Carrion V. G. Weems C. F. Eliez S. Patwardhan A. Brown W. Ray R. D.. (2001). Attenuation of frontal asymmetry in pediatric posttraumatic stress disorder. Biol. Psychiatry 50, 943–951. 10.1016/S0006-3223(01)01218-5 11750890

[B30] Chapman D. P. Whitfield C. L. Felitti V. J. Dube S. R. Edwards V. J. Anda R. F. (2004). Adverse childhood experiences and the risk of depressive disorders in adulthood. J. Affect. Disord. 82, 217–225. 10.1016/j.jad.2003.12.013 15488250

[B31] Chareyron L. J. Lavenex P. B. Lavenex P. (2012). Postnatal development of the amygdala: a stereological study in rats. J. Comp. Neurol. 520, 3745–3763. 10.1002/cne.23132 22523001

[B32] Chen E. Langer D. A. Raphaelson Y. E. Matthews K. A. (2004). Socioeconomic status and health in adolescents: the role of stress interpretations. Child Dev. 75, 1039–1052. 10.1111/j.1467-8624.2004.00724.x 15260863

[B33] Chen E. Matthews K. A. (2003). Development of the cognitive appraisal and understanding of social events (CAUSE) videos. Health Psychol. 22, 106–110. 10.1037/0278-6133.22.1.106 12558208

[B34] Chen E. Paterson L. Q. (2006). Neighborhood, family, and subjective socioeconomic status: how do they relate to adolescent health? Health Psychol. 25, 704–714. 10.1037/0278-6133.25.6.704 17100499

[B35] Cicchetti D. (2016). Socioemotional, personality, and biological development: illustrations from a multilevel developmental psychopathology perspective on child maltreatment. Annu. Rev. Psychol. 67, 187–211. 10.1146/annurev-psych-122414-033259 26726964

[B36] Cicchetti D. Ng R. (2014). Emotional development in maltreated children. Child. Emot. 26, 29–41. 10.1159/000354349

[B37] Cisler J. M. Privratsky A. A. Sartin-Tarm A. Sellnow K. Ross M. Weaver S.. (2020). l -DOPA and consolidation of fear extinction learning among women with posttraumatic stress disorder. Transl. Psychiatry 10, 1–11. 10.1038/s41398-020-00975-3 32801342 PMC7429959

[B38] Cohen M. M. Jing D. Yang R. R. Tottenham N. Lee F. S. Casey B. (2013). Early-life stress has persistent effects on amygdala function and development in mice and humans. Proc. Natl. Acad. Sci. U. S. A. 110, 18274–18278. 10.1073/pnas.1310163110 24145410 PMC3831447

[B39] Cohen R. A. Grieve S. Hoth K. F. Paul R. H. Sweet L. Tate D.. (2006). Early life stress and morphometry of the adult anterior cingulate cortex and caudate nuclei. Biol. Psychiatry 59, 975–982. 10.1016/j.biopsych.2005.12.016 16616722

[B40] Connell A. M. Goodman S. H. (2002). The association between psychopathology in fathers versus mothers and children's internalizing and externalizing behavior problems: a meta-analysis. Psychol. Bull. 128, 746–773. 10.1037/0033-2909.128.5.746 12206193

[B41] Conrad C. D. Galea L. A. M. Kuroda Y. McEwen B. S. (1996). Chronic stress impairs rat spatial memory on the Y maze, and this effect is blocked by tianeptine treatment. Behav. Neurosci. 110, 1321–1334. 10.1037/0735-7044.110.6.1321 8986335

[B42] Conrad C. D. Magariños A. M. LeDoux J. E. McEwen B. S. (1999). Repeated restraint stress facilitates fear conditioning independently of causing hippocampal CA3 dendritic atrophy. Behav. Neurosci. 113, 902–913. 10.1037/0735-7044.113.5.902 10571474

[B43] Copeland W. E. Shanahan L. Hinesley J. Chan R. F. Aberg K. A. Fairbank J. A.. (2018). Association of childhood trauma exposure with adult psychiatric disorders and functional outcomes. JAMA Netw. Open 1:e184493. 10.1001/jamanetworkopen.2018.4493 30646356 PMC6324370

[B44] Cortese B. M. Phan K. L. (2005). The role of glutamate in anxiety and related disorders. CNS Spectr. 10, 820–830. 10.1017/S1092852900010427 16400245

[B45] Crick N. R. Dodge K. A. (1994). A review and reformulation of social information-processing mechanisms in children's social adjustment. Psychol. Bull. 115, 74–101. 10.1037/0033-2909.115.1.74

[B46] Cunningham M. G. Bhattacharyya S. Benes F. M. (2002). Amygdalo-cortical sprouting continues into early adulthood: Implications for the development of normal and abnormal function during adolescence. J. Comp. Neurol. 453, 116–130. 10.1002/cne.10376 12373778

[B47] Cyr C. Euser E. bakermans-kranenburg M. van IJzendoorn M. (2010). Attachment security and disorganization in maltreating and high-risk families: a series of meta-analyses. Dev. Psychopathol. 22, 87–108. 10.1017/S0954579409990289 20102649

[B48] Danese A. McEwen B. S. (2012). Adverse childhood experiences, allostasis, allostatic load, and age-related disease. Physiol. Behav. 106, 29–39. 10.1016/j.physbeh.2011.08.019 21888923

[B49] Danese A. Widom C. S. (2020). Objective and subjective experiences of child maltreatment and their relationships with psychopathology. Nat. Hum. Behav. 3, 1–8. 10.1038/s41562-020-0880-3 32424258

[B50] Das R. K. Gale G. Walsh K. Hennessy V. E. Iskandar G. Mordecai L. A.. (2019). Ketamine can reduce harmful drinking by pharmacologically rewriting drinking memories. Nat. Commun. 10:5187. 10.1038/s41467-019-13162-w 31772157 PMC6879579

[B51] Davis M. Whalen P. J. (2001). The amygdala: vigilance and emotion. Mol. Psychiatry 6, 13–34. 10.1038/sj.mp.4000812 11244481

[B52] De Bellis M. D. Hall J. Boring A. M. Frustaci K. Moritz G. (2001). A pilot longitudinal study of hippocampal volumes in pediatric maltreatment-related posttraumatic stress disorder. Biol. Psychiatry 50, 305–309. 10.1016/S0006-3223(01)01105-2 11522266

[B53] De Bellis M. D. Keshavan M. S. Clark D. B. Casey B. J. Giedd J. N. Boring A. M.. (1999). A.E. Bennett Research Award. Developmental traumatology. Part II: brain development. Biol. Psychiatry 45, 1271–1284. 10.1016/S0006-3223(99)00045-1 10349033

[B54] De Bellis M. D. Keshavan M. S. Shifflett H. Iyengar S. Beers S. R. Hall J.. (2002). Brain structures in pediatric maltreatment-related posttraumatic stress disorder: a sociodemographically matched study. Biol. Psychiatry 52, 1066–1078. 10.1016/S0006-3223(02)01459-2 12460690

[B55] Debiec J. Doyere V. Nader K. LeDoux J. E. (2006). Directly reactivated, but not indirectly reactivated, memories undergo reconsolidation in the amygdala. Proc. Natl. Acad. Sci. U. S. A. 103, 3428–3433. 10.1073/pnas.0507168103 16492789 PMC1413871

[B56] Del-Ben C. M. Ferreira C. A. Q. Sanchez T. A. Alves-Neto W. C. Guapo V. G. de Araujo D. B.. (2012). Effects of diazepam on BOLD activation during the processing of aversive faces. J. Psychopharmacol. Oxf. Engl. 26, 443–451. 10.1177/0269881110389092 21106607

[B57] Dewey J. Hana G. Russell T. Price J. McCaffrey D. Harezlak J.. (2010). Reliability and validity of MRI-based automated volumetry software relative to auto-assisted manual measurement of subcortical structures in HIV-infected patients from a multisite study. NeuroImage 51, 1334–1344. 10.1016/j.neuroimage.2010.03.033 20338250 PMC2884380

[B58] Dodge K. A. (1993). Social-cognitive mechanisms in the development of conduct disorder and depression. Annu. Rev. Psychol. Palo Alto 44:559. 10.1146/annurev.ps.44.020193.003015 8434896

[B59] Dodge K. A. Bates J. E. Pettit G. S. (1990). Mechanisms in the cycle of violence. Science 250, 1678–1683. 10.1126/science.2270481 2270481

[B60] Do-Monte F. H. Quiñones-Laracuente K. Quirk G. J. (2015). A temporal shift in the circuits mediating retrieval of fear memory. Nature 519, 460–463. 10.1038/nature14030 25600268 PMC4376623

[B61] Dougherty L. R. Klein D. N. Davila J. (2004). A growth curve analysis of the course of dysthymic disorder: the effects of chronic stress and moderation by adverse parent-child relationships and family history. J. Consult. Clin. Psychol. 72:1012. 10.1037/0022-006X.72.6.1012 15612848

[B62] Driessen M. Herrmann J. Stahl K. Zwaan M. Meier S. Hill A.. (2000). Magnetic resonance imaging volumes of the hippocampus and the amygdala in women with borderline personality disorder and early traumatization. Arch. Gen. Psychiatry 57, 1115–1122. 10.1001/archpsyc.57.12.1115 11115325

[B63] Dube S. R. Anda R. F. Felitti V. J. Chapman D. P. Williamson D. F. Giles W. H. (2001). Childhood abuse, household dysfunction, and the risk of attempted suicide throughout the life span: findings from the Adverse Childhood Experiences Study. JAMA 286, 3089–3096. 10.1001/jama.286.24.3089 11754674

[B64] Dube S. R. Felitti V. J. Dong M. Chapman D. P. Giles W. H. Anda R. F. (2003). Childhood abuse, neglect, and household dysfunction and the risk of illicit drug use: the adverse childhood experiences study. Pediatrics 111, 564–572. 10.1542/peds.111.3.564 12612237

[B65] Duvarci S. Pare D. (2014). Amygdala microcircuits controlling learned fear. Neuron 82, 966–980. 10.1016/j.neuron.2014.04.042 24908482 PMC4103014

[B66] Edmiston E. E. Wang F. Mazure C. M. Guiney J. Sinha R. Mayes L. C.. (2011). Corticostriatal-limbic gray matter morphology in adolescents with self-reported exposure to childhood maltreatment. Arch. Pediatr. Adolesc. Med. 165, 1069–1077. 10.1001/archpediatrics.2011.565 22147775 PMC3607102

[B67] Ellis B. J. Abrams L. S. Masten A. S. Sternberg R. J. Tottenham N. Frankenhuis W. E. (2021). Hidden talents in harsh environments. Dev. Psychopathol. 887, 1–19. 10.1017/S0954579420000887 32672144

[B68] Ellis B. J. Bianchi J. Griskevicius V. Frankenhuis W. E. (2017). Beyond risk and protective factors: an adaptation-based approach to resilience. Perspect. Psychol. Sci. 12, 561–587. 10.1177/1745691617693054 28679332

[B69] Ellwood-Lowe M. E. Humphreys K. L. Ordaz S. J. Camacho M. C. Sacchet M. D. Gotlib I. H. (2018). Time-varying effects of income on hippocampal volume trajectories in adolescent girls. Dev. Cogn. Neurosci. 30, 41–50. 10.1016/j.dcn.2017.12.005 29275097 PMC5963716

[B70] Emery R. E. Laumann-Billings L. (1998). An overview of the nature, causes, and consequences of abusive family relationships: toward differentiating maltreatment and violence. Am. Psychol. 53, 121–135. 10.1037/0003-066X.53.2.121 9491743

[B71] Espejo E. P. Hammen C. L. Connolly N. P. Brennan P. A. Najman J. M. Bor W. (2007). Stress sensitization and adolescent depressive severity as a function of childhood adversity: a link to anxiety disorders. J. Abnorm. Child Psychol. 35, 287–299. 10.1007/s10802-006-9090-3 17195949

[B72] Etkin A. Wager T. D. (2007). Functional neuroimaging of anxiety: a meta-ana lysis of emotional processing in PTSD, social anxiety disorder, and specific phobia. Am. J. Psychiatry 164, 1476–1488. 10.1176/appi.ajp.2007.07030504 17898336 PMC3318959

[B73] Evans G. W. Cassells R. C. (2014). Childhood poverty, cumulative risk exposure, and mental health in emerging adults. Clin. Psychol. Sci. 2, 287–296. 10.1177/2167702613501496 26609499 PMC4655888

[B74] Evans G. W. English K. (2002). The environment of poverty: multiple stressor exposure, psychophysiological stress, and socioemotional adjustment. Child Dev. 73, 1238–1248. 10.1111/1467-8624.00469 12146745

[B75] Evans G. W. Swain J. E. King A. P. Wang X. Javanbakht A. Ho S. S.. (2016). Childhood cumulative risk exposure and adult amygdala volume and function. J. Neurosci. Res. 94, 535–543. 10.1002/jnr.23681 26469872 PMC4833698

[B76] Evans S. E. Davies C. DiLillo D. (2008). Exposure to domestic violence: a meta-analysis of child and adolescent outcomes. Aggress. Violent Behav. 13, 131–140. 10.1016/j.avb.2008.02.005

[B77] Farber M. J. Gee D. G. Hariri A. R. (2020). Normative range parenting and the developing brain: a scoping review and recommendations for future research. Eur. J. Neurosci. 2020:ejn.15003. 10.1111/ejn.15003 33051903 PMC8044268

[B78] Felitti V. J. Anda R. F. Nordenberg D. Williamson D. F. Spitz A. M. Edwards V.. (1998). Relationship of childhood abuse and household dysfunction to many of the leading causes of death in adults. Am. J. Prev. Med. 14, 245–258. 10.1016/S0749-3797(98)00017-8 9635069

[B79] Finnema S. J. Nabulsi N. B. Eid T. Detyniecki K. Lin S. Chen M.-K.. (2016). Imaging synaptic density in the living human brain. Sci. Transl. Med. 8:348ra96. 10.1126/scitranslmed.aaf6667 27440727

[B80] Fox A. S. Oler J. A. Tromp D. P. Fudge J. L. Kalin N. H. (2015). Extending the amygdala in theories of threat processing. Trends Neurosci. 38, 319–329. 10.1016/j.tins.2015.03.002 25851307 PMC4417372

[B81] Frankenhuis W. E. Young E. S. Ellis B. J. (2020). The hidden talents approach: theoretical and methodological challenges. Trends Cogn. Sci. 24, 569–581. 10.1016/j.tics.2020.03.007 32360117

[B82] Frodl T. Meisenzahl E. M. Zetzsche T. Born C. Jäger M. Groll C.. (2003). Larger amygdala volumes in first depressive episode as compared to recurrent major depression and healthy control subjects. Biol. Psychiatry 53, 338–344. 10.1016/S0006-3223(02)01474-9 12586453

[B83] García-Amado M. Prensa L. (2013). Distribution of dopamine transporter immunoreactive fibers in the human amygdaloid complex. Eur. J. Neurosci. 38, 3589–3601. 10.1111/ejn.12358 24102648

[B84] Gerritsen L. Kalpouzos G. Westman E. Simmons A. Wahlund L. O. Bäckman L.. (2015). The influence of negative life events on hippocampal and amygdala volumes in old age: a life-course perspective. Psychol. Med. 45, 1219–1228. 10.1017/S0033291714002293 25273347

[B85] Ghosh S. Chattarji S. (2015). Neuronal encoding of the switch from specific to generalized fear. Nat. Neurosci. 18, 112–120. 10.1038/nn.3888 25436666

[B86] Goddings A. L. Mills K. L. Clasen L. S. Giedd J. N. Viner R. M. Blakemore S. J. (2014). The influence of puberty on subcortical brain development. NeuroImage. 10.1016/j.neuroimage.2013.09.073 24121203 PMC3991320

[B87] Gold A. L. Sheridan M. A. Peverill M. Busso D. S. Lambert H. K. Alves S.. (2016). Childhood abuse and reduced cortical thickness in brain regions involved in emotional processing. J. Child Psychol. Psychiatry 57, 1154–1164. 10.1111/jcpp.12630 27647051 PMC5031358

[B88] Gorka A. X. Hanson J. L. Radtke S. R. Hariri A. R. (2014). Reduced hippocampal and medial prefrontal gray matter mediate the association between reported childhood maltreatment and trait anxiety in adulthood and predict sensitivity to future life stress. Biol. Mood Anxiety Disord. 4:12. 10.1186/2045-5380-4-12 25408863 PMC4236295

[B89] Guastella A. J. Richardson R. Lovibond P. F. Rapee R. M. Gaston J. E. Mitchell P.. (2008). A randomized controlled trial of D-cycloserine enhancement of exposure therapy for social anxiety disorder. Biol. Psychiatry 63, 544–549. 10.1016/j.biopsych.2007.11.011 18179785

[B90] Haaker J. Gaburro S. Sah A. Gartmann N. Lonsdorf T. B. Meier K.. (2013). Single dose of l-dopa makes extinction memories context-independent and prevents the return of fear. Proc. Natl. Acad. Sci. U. S. A. 110, E2428–E2436. 10.1073/pnas.1303061110 23754384 PMC3696794

[B91] Hadad-Ophir O. Albrecht A. Stork O. Richter-Levin G. (2014). Amygdala activation and GABAergic gene expression in hippocampal sub-regions at the interplay of stress and spatial learning. Front. Behav. Neurosci. 8:3. 10.3389/fnbeh.2014.00003 24478650 PMC3896990

[B92] Hair N. L. Hanson J. L. Wolfe B. L. Pollak S. D. (2015). Association of child poverty, brain development, and academic achievement. JAMA Pediatr. 169, 822–829. 10.1001/jamapediatrics.2015.1475 26192216 PMC4687959

[B93] Hamilton J. P. Etkin A. Furman D. J. Lemus M. G. Johnson R. F. Gotlib I. H. (2012). Functional neuroimaging of major depressive disorder: a meta-analysis and new integration of baseline activation and neural response data. Am. J. Psychiatry 169, 693–703. 10.1176/appi.ajp.2012.11071105 22535198 PMC11889638

[B94] Hammen C. (2018). Risk factors for depression: an autobiographical review. Annu. Rev. Clin. Psychol. 14, 1–28. 10.1146/annurev-clinpsy-050817-084811 29328780

[B95] Hammen C. Henry R. Daley S. E. (2000). Depression and sensitization to stressors among young women as a function of childhood adversity. J. Consult. Clin. Psychol. 68, 782–787. 10.1037/0022-006X.68.5.782 11068964

[B96] Hanson J. L. Albert D. Skinner A. T. Shen S. H. Dodge K. A. Lansford J. E. (2019). Resting state coupling between the amygdala and ventromedial prefrontal cortex is related to household income in childhood and indexes future psychological vulnerability to stress. Dev. Psychopathol. 31:1053. 10.1017/S0954579419000592 31084654

[B97] Hanson J. L. Chung M. K. Avants B. B. Rudolph K. D. Shirtcliff E. A. Gee J. C.. (2012a). Structural variations in prefrontal cortex mediate the relationship between early childhood stress and spatial working memory. J. Neurosci. 32, 7917–7925. 10.1523/JNEUROSCI.0307-12.2012 22674267 PMC3375595

[B98] Hanson J. L. Knodt A. R. Brigidi B. D. Hariri A. R. (2015a). Lower structural integrity of the uncinate fasciculus is associated with a history of child maltreatment and future psychological vulnerability to stress. Dev. Psychopathol. 27:1611. 10.1017/S0954579415000978 26535947 PMC4698331

[B99] Hanson J. L. Knodt A. R. Brigidi B. D. Hariri A. R. (2018). Heightened connectivity between the ventral striatum and medial prefrontal cortex as a biomarker for stress-related psychopathology: understanding interactive effects of early and more recent stress. Psychol. Med. 48:1835. 10.1017/S0033291717003348 29248021 PMC6301079

[B100] Hanson J. L. Nacewicz B. M. Sutterer M. J. Cayo A. A. Schaefer S. M. Rudolph K. D.. (2015b). Behavioral problems after early life stress: contributions of the hippocampus and amygdala. Biol. Psychiatry 77, 314–323. 10.1016/j.biopsych.2014.04.020 24993057 PMC4241384

[B101] Hanson J. L. Suh J. W. Nacewicz B. M. Sutterer M. J. Cayo A. A. Stodola D. E.. (2012b). Robust automated amygdala segmentation *via* multi-atlas diffeomorphic registration. Front. Neurosci. 6:166. 10.3389/fnins.2012.00166 23226114 PMC3509347

[B102] Hariri A. R. (2009). The neurobiology of individual differences in complex behavioral traits. Annu. Rev. Neurosci. 32, 225–247. 10.1146/annurev.neuro.051508.135335 19400720 PMC2755193

[B103] Harkness K. L. Bruce A. E. Lumley M. N. (2006). The role of childhood abuse and neglect in the sensitization to stressful life events in adolescent depression. J. Abnorm. Psychol. 115:730. 10.1037/0021-843X.115.4.730 17100530

[B104] Herry C. Bach D. R. Esposito F. Salle F. D. Perrig W. J. Scheffler K.. (2007). Processing of temporal unpredictability in human and animal amygdala. J. Neurosci. 27, 5958–5966. 10.1523/JNEUROSCI.5218-06.2007 17537966 PMC6672268

[B105] Herry C. Ciocchi S. Senn V. Demmou L. Müller C. Lüthi A. (2008). Switching on and off fear by distinct neuronal circuits. Nature 454, 600–606. 10.1038/nature07166 18615015

[B106] Herzog J. I. Thome J. Demirakca T. Koppe G. Ende G. Lis S.. (2020). Influence of severity of type and timing of retrospectively reported childhood maltreatment on female amygdala and hippocampal volume. Sci. Rep. 10:57490. 10.1038/s41598-020-57490-0 32024861 PMC7002661

[B107] Hetzel A. Rosenkranz J. A. (2014). Distinct effects of repeated restraint stress on basolateral amygdala neuronal membrane properties in resilient adolescent and adult rats. Neuropsychopharmacology 39, 2114–2130. 10.1038/npp.2014.60 24619244 PMC4104329

[B108] Hodel A. S. Hunt R. H. Cowell R. A. Van Den Heuvel S. E. Gunnar M. R. Thomas K. M. (2015). Duration of early adversity and structural brain development in post-institutionalized adolescents. NeuroImage 105, 112–119. 10.1016/j.neuroimage.2014.10.020 25451478 PMC4262668

[B109] Hooley J. M. Gotlib I. H. (2000). A diathesis-stress conceptualization of expressed emotion and clinical outcome. Appl. Prev. Psychol. 9, 135–151. 10.1016/S0962-1849(05)80001-0

[B110] Humphrey T. (1968). The development of the human amygdala during early embryonic life. J. Comp. Neurol. 132, 135–165. 10.1002/cne.901320108 5732427

[B111] Infurna F. J. Jayawickreme E. (2019). Fixing the growth illusion: new directions for research in resilience and posttraumatic growth. Curr. Dir. Psychol. Sci. 28, 152–158. 10.1177/0963721419827017

[B112] Irle E. Lange C. Sachsse U. Weniger G. (2009). Further evidence that post-traumatic stress disorder but not dissociative disorders are related to amygdala and hippocampal size reduction in trauma-exposed individuals. Acta Psychiatr. Scand. 119, 330–331. 10.1111/j.1600-0447.2009.01351.x 19207126

[B113] Jacobson-Pick S. Elkobi A. Vander S. Rosenblum K. Richter-Levin G. (2008). Juvenile stress-induced alteration of maturation of the GABAA receptor α subunit in the rat. Int. J. Neuropsychopharmacol. 11, 891–903. 10.1017/S1461145708008559 18364065

[B114] Jacobson-Pick S. Richter-Levin G. (2012). Short- and long-term effects of juvenile stressor exposure on the expression of GABAA receptor subunits in rats. Stress 15, 416–424. 10.3109/10253890.2011.634036 22044189

[B115] Jaffee S. R. (2017). Child maltreatment and risk for psychopathology in childhood and adulthood. Annu. Rev. Clin. Psychol. 13, 525–551. 10.1146/annurev-clinpsy-032816-045005 28375720

[B116] Jurkowski M. P. Bettio L. Woo E. K. Patten A. Yau S. Y. Gil-Mohapel J. (2020). Beyond the hippocampus and the SVZ: adult neurogenesis throughout the brain. Front. Cell. Neurosci. 14:293. 10.3389/fncel.2020.576444 33132848 PMC7550688

[B117] Kalisch R. Holt B. Petrovic P. De Martino B. Klöppel S. Büchel C.. (2009). The NMDA agonist D-cycloserine facilitates fear memory consolidation in humans. Cereb. Cortex 19, 187–196. 10.1093/cercor/bhn076 18477687 PMC2638747

[B118] Kanner A. D. Coyne J. C. Schaefer C. Lazarus R. S. (1981). Comparison of two modes of stress measurement: daily hassles and uplifts versus major life events. J. Behav. Med. 4, 1–39. 10.1007/BF00844845 7288876

[B119] Karl A. Schaefer M. Malta L. S. Dörfel D. Rohleder N. Werner A. (2006). A meta-analysis of structural brain abnormalities in PTSD. Neurosci. Biobehav. Rev. 30, 1004–1031. 10.1016/j.neubiorev.2006.03.004 16730374

[B120] Kellogg N. D. Menard S. W. (2003). Violence among family members of children and adolescents evaluated for sexual abuse. Child Abuse Negl. 27, 1367–1376. 10.1016/j.chiabu.2003.10.008 14644055

[B121] Kendler K. S. Kuhn J. W. Prescott C. A. (2004). Childhood sexual abuse, stressful life events and risk for major depression in women. Psychol. Med. 34:1475. 10.1017/S003329170400265X 15724878

[B122] Kessler R. C. McLaughlin K. A. Green J. G. Gruber M. J. Sampson N. A. Zaslavsky A. M.. (2010). Childhood adversities and adult psychopathology in the WHO world mental health surveys. Br. J. Psychiatry 197, 378–385. 10.1192/bjp.bp.110.080499 21037215 PMC2966503

[B123] Kim H. Somerville L. H. Johnstone T. Alexander A. L. Whalen P. J. (2003). Inverse amygdala and medial prefrontal cortex responses to surprised faces. NeuroReport 14, 2317–2322. 10.1097/00001756-200312190-00006 14663183

[B124] Kim J. E. Lyoo I. K. Estes A. M. Renshaw P. F. Shaw D. W. Friedman S. D.. (2010). Laterobasal amygdalar enlargement in 6- to 7-year-old children with autism spectrum disorder. Arch. Gen. Psychiatry. 67, 1187–1197. 10.1001/archgenpsychiatry.2010.148 21041620

[B125] Kim Y. Sakata H. Nejime M. Konoike N. Miyachi S. Nakamura K. (2018). Afferent connections of the dorsal, perigenual, and subgenual anterior cingulate cortices of the monkey: amygdalar inputs and intrinsic connections. Neurosci. Lett. 681, 93–99. 10.1016/j.neulet.2018.05.028 29803854

[B126] Kindt M. Soeter M. Vervliet B. (2009). Beyond extinction: erasing human fear responses and preventing the return of fear. Nat. Neurosci. 12, 256–258. 10.1038/nn.2271 19219038

[B127] King L. S. Humphreys K. L. Camacho M. C. Gotlib I. H. (2019). A person-centered approach to the assessment of early life stress: associations with the volume of stress-sensitive brain regions in early adolescence. Dev. Psychopathol. 31, 643–655. 10.1017/S0954579418000184 29716668 PMC6214790

[B128] Kuo J. R. Kaloupek D. G. Woodward S. H. (2012). Amygdala volume in combat-exposed veterans with and without posttraumatic stress disorder: a cross-sectional study. Arch. Gen. Psychiatry 69:1080. 10.1001/archgenpsychiatry.2012.73 23026958

[B129] Lanteaume L. Khalfa S. Régis J. Marquis P. Chauvel P. Bartolomei F. (2007). Emotion induction after direct intracerebral stimulations of human amygdala. Cereb. Cortex 17, 1307–1313. 10.1093/cercor/bhl041 16880223

[B130] Lazarus R. S. Folkman S. (1987). Transactional theory and research on emotions and coping. Eur. J. Personal. 1, 141–169. 10.1002/per.2410010304

[B131] Letourneau N. L. Duffett-Leger L. Levac L. Watson B. Young-Morris C. (2013). Socioeconomic status and child development: a meta-analysis. J. Emot. Behav. Disord. 21, 211–224. 10.1177/1063426611421007

[B132] Liem J. H. Boudewyn A. C. (1999). Contextualizing the effects of childhood sexual abuse on adult self- and social functioning: an attachment theory perspective. Child Abuse Negl. 23, 1141–1157. 10.1016/S0145-2134(99)00081-2 10604068

[B133] Likhtik E. Popa D. Apergis-Schoute J. Fidacaro G. A. Paré D. (2008). Amygdala intercalated neurons are required for expression of fear extinction. Nature 454, 642–645. 10.1038/nature07167 18615014 PMC2528060

[B134] Lindquist K. A. Wager T. D. Kober H. Bliss-Moreau E. Barrett L. F. (2012). The brain basis of emotion: a meta-analytic review. Behav. Brain Sci. 35, 121–143. 10.1017/S0140525X11000446 22617651 PMC4329228

[B135] Liu Y. Nacewicz B. M. Zhao G. Adluru N. Kirk G. R. Ferrazzano P. A.. (2020). A 3D fully convolutional neural network with top-down attention-guided refinement for accurate and robust automatic segmentation of amygdala and its subnuclei. Front. Neurosci. 14:260. 10.3389/fnins.2020.00260 32508558 PMC7253589

[B136] Liu Z.-P. Song C. Wang M. He Y. Xu X.-B. Pan H.-Q.. (2014). Chronic stress impairs GABAergic control of amygdala through suppressing the tonic GABAA receptor currents. Mol. Brain 7:32. 10.1186/1756-6606-7-32 24758222 PMC4012764

[B137] Llorente M. A. G. Charlebois L. M.-M. Ducci V. Farias A. M. (2003). Children in Institutions: The Beginning of the End? The Cases of Italy, Spain, Argentina, Chile and Uruguay. Innocenti Insight. Piazza, SS: UNICEF Innocenti Research Centre.

[B138] Logan J. (2011). Suicide categories by patterns of known risk factors: a latent class analysis. Arch. Gen. Psychiatry 68:935. 10.1001/archgenpsychiatry.2011.85 21893660

[B139] LoPilato A. M. Goines K. Addington J. Bearden C. E. Cadenhead K. S. Cannon T. D.. (2019). Impact of childhood adversity on corticolimbic volumes in youth at clinical high-risk for psychosis. Schizophr. Res. 213, 48–55. 10.1016/j.schres.2019.01.048 30745068

[B140] Luby J. Belden A. Botteron K. Marrus N. Harms M. P. Babb C.. (2013). The effects of poverty on childhood brain development: the mediating effect of caregiving and stressful life events. JAMA Pediatr. 167:1135. 10.1001/jamapediatrics.2013.3139 24165922 PMC4001721

[B141] Magariños A. M. McEwen B. S. Flügge G. Fuchs E. (1996). Chronic psychosocial stress causes apical dendritic atrophy of hippocampal CA3 pyramidal neurons in subordinate tree shrews. J. Neurosci. 16, 3534–3540. 10.1523/JNEUROSCI.16-10-03534.1996 8627386 PMC6579123

[B142] Magariños A. M. Verdugo J. M. G. McEwen B. S. (1997). Chronic stress alters synaptic terminal structure in hippocampus. Proc. Natl. Acad. Sci. U. S. A. 94, 14002–14008. 10.1073/pnas.94.25.14002 9391142 PMC28422

[B143] Mahan A. L. Ressler K. J. (2012). Fear conditioning, synaptic plasticity and the amygdala: implications for posttraumatic stress disorder. Trends Neurosci. 35, 24–35. 10.1016/j.tins.2011.06.007 21798604 PMC3206195

[B144] Maier S. F. Seligman M. E. (1976). Learned helplessness: theory and evidence. J. Exp. Psychol. Gen. 105, 3–46. 10.1037/0096-3445.105.1.3

[B145] Maier S. F. Watkins L. R. (2005). Stressor controllability and learned helplessness: the roles of the dorsal raphe nucleus, serotonin, and corticotropin-releasing factor. Neurosci. Biobehav. Rev. 29, 829–841. 10.1016/j.neubiorev.2005.03.021 15893820

[B146] Mallett X. Schall U. (2019). The psychological and physiological sequel of child maltreatment: a forensic perspective. Neurol. Psychiatry Brain Res. 34, 9–12. 10.1016/j.npbr.2019.08.003

[B147] Marowsky A. Yanagawa Y. Obata K. Vogt K. E. (2005). A specialized subclass of interneurons mediates dopaminergic facilitation of amygdala function. Neuron 48, 1025–1037. 10.1016/j.neuron.2005.10.029 16364905

[B148] Masten A. S. Hubbard J. J. Gest S. D. Tellegen A. Garmezy N. Ramirez M. (1999). Competence in the context of adversity: pathways to resilience and maladaptation from childhood to late adolescence. Dev. Psychopathol. 11, 143–169. 10.1017/S0954579499001996 10208360

[B149] Masten A. S. Lucke C. M. Nelson K. M. Stallworthy I. C. (2021). Resilience in development and psychopathology: multisystem perspectives. Annu. Rev. Clin. Psychol. 17. 10.1146/annurev-clinpsy-081219-120307 33534615

[B150] McCall R. B. Groark C. J. Fish L. Muhamedrahimov R. J. Palmov O. I. Nikiforova N. V. (2013). Maintaining a social-emotional intervention and its benefits for institutionalized children. Child Dev. 84, 1734–1749. 10.1111/cdev.12098 23551051 PMC3706532

[B151] McCall R. B. Groark C. J. Hawk B. N. Julian M. M. Merz E. C. Rosas J. M.. (2019). Early caregiver–child interaction and children's development: lessons from the St. Petersburg-USA orphanage intervention research project. Clin. Child Family Psychol. Rev. 22, 208–224. 10.1007/s10567-018-0270-9 30196471

[B152] McEwen B. S. (1998). Protective and damaging effects of stress mediators. N. Engl. J. Med. 338, 171–179. 10.1056/NEJM199801153380307 9428819

[B153] McEwen B. S. (2003). Mood disorders and allostatic load. Biol. Psychiatry 54, 200–207. 10.1016/S0006-3223(03)00177-X 12893096

[B154] McEwen B. S. (2005). Glucocorticoids, depression, and mood disorders: structural remodeling in the brain. Metabolism 54, 20–23. 10.1016/j.metabol.2005.01.008 15877308

[B155] McEwen B. S. Bowles N. P. Gray J. D. Hill M. N. Hunter R. G. Karatsoreos I. N.. (2015). Mechanisms of stress in the brain. Nat. Neurosci. 18, 1353–1363. 10.1038/nn.4086 26404710 PMC4933289

[B156] McLaughlin K. A. Breslau J. Green J. G. Lakoma M. D. Sampson N. A. Zaslavsky A. M.. (2011). Childhood socio-economic status and the onset, persistence, and severity of DSM-IV mental disorders in a US national sample. Soc. Sci. Med. 73, 1088–1096. 10.1016/j.socscimed.2011.06.011 21820781 PMC3191493

[B157] McLaughlin K. A. Conron K. J. Koenen K. C. Gilman S. E. (2010). Childhood adversity, adult stressful life events, and risk of past-year psychiatric disorder: a test of the stress sensitization hypothesis in a population-based sample of adults. Psychol. Med. 40:1647. 10.1017/S0033291709992121 20018126 PMC2891275

[B158] McLaughlin K. A. Sheridan M. Humphreys K. L. Belsky J. Ellis B. J. (2020). The value of dimensional models of early experience: thinking clearly about concepts and categories. PsyArXiv. 10.31234/osf.io/29fmt PMC8563369 34491864

[B159] McLaughlin K. A. Sheridan M. A. Gold A. L. Duys A. Lambert H. K. Peverill M.. (2016). Maltreatment exposure, brain structure, and fear conditioning in children and adolescents. Neuropsychopharmacology 41, 1956–1964. 10.1038/npp.2015.365 26677946 PMC4908632

[B160] McLaughlin K. A. Sheridan M. A. Lambert H. K. (2014a). Childhood adversity and neural development: deprivation and threat as distinct dimensions of early experience. Neurosci. Biobehav. Rev. 47, 578–591. 10.1016/j.neubiorev.2014.10.012 25454359 PMC4308474

[B161] McLaughlin K. A. Sheridan M. A. Winter W. Fox N. A. Zeanah C. H. Nelson C. A. (2014b). Widespread reductions in cortical thickness following severe early-life deprivation: a neurodevelopmental pathway to attention-deficit/hyperactivity disorder. Biol. Psychiatry 76, 629–638. 10.1016/j.biopsych.2013.08.016 24090797 PMC3969891

[B162] Mehta M. A. Golembo N. I. Nosarti C. Colvert E. Mota A. Williams S. C.. (2009). Amygdala, hippocampal and corpus callosum size following severe early institutional deprivation: the English and Romanian Adoptees study pilot. J. Child Psychol. Psychiatry 50, 943–951. 10.1111/j.1469-7610.2009.02084.x 19457047

[B163] Merz E. C. Tottenham N. Noble K. G. (2018). Socioeconomic status, amygdala volume, and internalizing symptoms in children and adolescents. J. Clin. Child Adolesc. Psychol. 47, 312–323. 10.1080/15374416.2017.1326122 28574722 PMC6116521

[B164] Mitra R. Jadhav S. McEwen B. S. Vyas A. Chattarji S. (2005). Stress duration modulates the spatiotemporal patterns of spine formation in the basolateral amygdala. Proc. Natl. Acad. Sci. U. S. A. 102, 9371–9376. 10.1073/pnas.0504011102 15967994 PMC1166638

[B165] Monroe S. M. Harkness K. L. (2005). Life stress, the “kindling” hypothesis, and the recurrence of depression: considerations from a life stress perspective. Psychol. Rev. 112:417. 10.1037/0033-295X.112.2.417 15783292

[B166] Morey R. A. Haswell C. C. Hooper S. R. De Bellis M. D. (2016). Amygdala, hippocampus, and ventral medial prefrontal cortex volumes differ in maltreated youth with and without chronic posttraumatic stress disorder. Neuropsychopharmacology 41, 791–801. 10.1038/npp.2015.205 26171720 PMC4707825

[B167] Morey R. A. Petty C. M. Xu Y. Pannu Hayes J. Wagner H. R. Lewis D. V.. (2009). A comparison of automated segmentation and manual tracing for quantifying hippocampal and amygdala volumes. NeuroImage 45, 855–866. 10.1016/j.neuroimage.2008.12.033 19162198 PMC2714773

[B168] Moriceau S. Sullivan R. M. (2006). Maternal presence serves as a switch between learning fear and attraction in infancy. Nat. Neurosci. 9, 1004–1006. 10.1038/nn1733 16829957 PMC1560090

[B169] Mosconi M. W. Cody-Hazlett H. Poe M. D. Gerig G. Gimpel-Smith R. Piven J. (2009). Longitudinal study of amygdala volume and joint attention in 2- to 4-year-old children with autism. Arch. Gen. Psychiatry. 66, 509–516. 10.1001/archgenpsychiatry.2009.19 19414710 PMC3156446

[B170] Moylan C. A. Herrenkohl T. I. Sousa C. Tajima E. A. Herrenkohl R. C. Russo M. J. (2010). The effects of child abuse and exposure to domestic violence on adolescent internalizing and externalizing behavior problems. J. Fam. Viol. 25, 53–63. 10.1007/s10896-009-9269-9 20495613 PMC2872483

[B171] Mozhui K. Karlsson R.-M. Kash T. L. Ihne J. Norcross M. Patel S.. (2010). Strain differences in stress responsivity are associated with divergent amygdala gene expression and glutamate-mediated neuronal excitability. J. Neurosci. 30, 5357–5367. 10.1523/JNEUROSCI.5017-09.2010 20392957 PMC2866495

[B172] Müller R. Kenney M. (2020). A science of hope? tracing emergent entanglements between the biology of early life adversity, trauma-informed care, and restorative justice. Sci. Technol. Hum. Values 2020:0162243920974095. 10.1177/0162243920974095

[B173] Muniz C. N. Fox B. Miley L. N. Delisi M. Cigarran G. P. Birnbaum A. (2019). The effects of adverse childhood experiences on internalizing versus externalizing outcomes. Crim. Justice Behav. 46, 568–589. 10.1177/0093854819826213

[B174] Nacewicz B. M. Angelos L. Dalton K. M. Fischer R. Anderle M. J. Alexander A. L.. (2012). Reliable non-invasive measurement of human neurochemistry using proton spectroscopy with an anatomically defined amygdala-specific voxel. NeuroImage 59, 2548–2559. 10.1016/j.neuroimage.2011.08.090 21924361 PMC3254833

[B175] Nacewicz B. M. Dalton K. M. Johnstone T. Long M. T. McAuliff E. M. Oakes T. R.. (2006). Amygdala volume and nonverbal social impairment in adolescent and adult males with autism. Arch. Gen. Psychiatry. 63, 1417–1428. 10.1001/archpsyc.63.12.1417 17146016 PMC4767012

[B176] Nader K. Schafe G. E. Le Doux J. E. (2000). Fear memories require protein synthesis in the amygdala for reconsolidation after retrieval. Nature 406, 722–726. 10.1038/35021052 10963596

[B177] Nanni V. Uher R. Danese A. (2012). Childhood maltreatment predicts unfavorable course of illness and treatment outcome in depression: a meta-analysis. Am. J. Psychiatry 169, 141–151. 10.1176/appi.ajp.2011.11020335 22420036

[B178] Nelson C. A. Gabard-Durnam L. J. (2020). Early adversity and critical periods: neurodevelopmental consequences of violating the expectable environment. Trends Neurosci. 43, 133–143. 10.1016/j.tins.2020.01.002 32101708 PMC8092448

[B179] Nibuya M. Takahashi M. Russell D. S. Duman R. S. (1999). Repeated stress increases catalytic TrkB mRNA in rat hippocampus. Neurosci. Lett. 267, 81–84. 10.1016/S0304-3940(99)00335-3 10400217

[B180] Noble K. G. Houston S. M. Kan E. Sowell E. R. (2012). Neural correlates of socioeconomic status in the developing human brain. Dev. Sci. 15, 516–527. 10.1111/j.1467-7687.2012.01147.x 22709401 PMC6554027

[B181] Odgers C. L. Jaffee S. R. (2013). Routine versus catastrophic influences on the developing child. Annu. Rev. Public Health 34, 29–48. 10.1146/annurev-publhealth-031912-114447 23297656 PMC4212823

[B182] Opendak M. Robinson-Drummer P. Blomkvist A. Zanca R. M. Wood K. Jacobs L.. (2019). Neurobiology of maternal regulation of infant fear: the role of mesolimbic dopamine and its disruption by maltreatment. Neuropsychopharmacology 44, 1247–1257. 10.1038/s41386-019-0340-9 30758321 PMC6784970

[B183] Otto M. W. Tolin D. F. Simon N. M. Pearlson G. D. Basden S. Meunier S. A.. (2010). Efficacy of D-cycloserine for enhancing response to cognitive-behavior therapy for panic disorder. Biol. Psychiatry 67, 365–370. 10.1016/j.biopsych.2009.07.036 19811776

[B184] Padival M. Quinette D. Rosenkranz J. A. (2013). Effects of repeated stress on excitatory drive of basal amygdala neurons *in vivo*. Neuropsychopharmacology 38, 1748–1762. 10.1038/npp.2013.74 23535779 PMC3717551

[B185] Padival M. A. Blume S. R. Vantrease J. E. Rosenkranz J. A. (2015). Qualitatively different effect of repeated stress during adolescence on principal neuron morphology across lateral and basal nuclei of the rat amygdala. Neuroscience 291, 128–145. 10.1016/j.neuroscience.2015.02.012 25701125 PMC4369409

[B186] Patton M. H. Bizup B. T. Grace A. A. (2013). The infralimbic cortex bidirectionally modulates mesolimbic dopamine neuron activity *via* distinct neural pathways. J. Neurosci. 33, 16865–16873. 10.1523/JNEUROSCI.2449-13.2013 24155293 PMC3807020

[B187] Paulus M. P. Feinstein J. S. Castillo G. Simmons A. N. Stein M. B. (2005). Dose-dependent decrease of activation in bilateral amygdala and insula by lorazepam during emotion processing. Arch. Gen. Psychiatry 62, 282–288. 10.1001/archpsyc.62.3.282 15753241

[B188] Penzo M. A. Robert V. Tucciarone J. De Bundel D. Wang M. Van Aelst L.. (2015). The paraventricular thalamus controls a central amygdala fear circuit. Nature 519, 455–459. 10.1038/nature13978 25600269 PMC4376633

[B189] Peverill M. Dirks M. A. Narvaja T. Herts K. L. Comer J. S. McLaughlin K. A. (2020). Socioeconomic status and child psychopathology in the United States: a meta-analysis of population-based studies. Clin. Psychol. Rev. 2020:101933. 10.1016/j.cpr.2020.101933 33278703 PMC7855901

[B190] Phelps E. A. (2004). Human emotion and memory: interactions of the amygdala and hippocampal complex. Curr. Opin. Neurobiol. 14, 198–202. 10.1016/j.conb.2004.03.015 15082325

[B191] Piotrowska P. J. Stride C. B. Croft S. E. Rowe R. (2015). Socioeconomic status and antisocial behaviour among children and adolescents: a systematic review and meta-analysis. Clin. Psychol. Rev. 35, 47–55. 10.1016/j.cpr.2014.11.003 25483561

[B192] Pittenger C. Sanacora G. Krystal J. H. (2007). The NMDA receptor as a therapeutic target in major depressive disorder. CNS Neurol. Disord. 6, 101–115. 10.2174/187152707780363267 17430148

[B193] Pitts-Taylor V. (2019). Neurobiologically poor? brain phenotypes, inequality, and biosocial determinism. Sci. Technol. Hum. Values 44, 660–685. 10.1177/0162243919841695

[B194] Pollak S. D. Cicchetti D. Hornung K. Reed A. (2000). Recognizing emotion in faces: developmental effects of child abuse and neglect. Dev. Psychol. 36, 679–688. 10.1037/0012-1649.36.5.679 10976606

[B195] Pollak S. D. Sinha P. (2002). Effects of early experience on children's recognition of facial displays of emotion. Dev. Psychol. 38, 784–791. 10.1037/0012-1649.38.5.784 12220055

[B196] Pollak S. D. Tolley-Schell S. A. (2003). Selective attention to facial emotion in physically abused children. J. Abnorm. Psychol. 112, 323–338. 10.1037/0021-843X.112.3.323 12943012

[B197] Przybyslawski J. Roullet P. Sara S. J. (1999). Attenuation of emotional and nonemotional memories after their reactivation: role of beta adrenergic receptors. J. Neurosci. Off. J. Soc. Neurosci. 19, 6623–6628. 10.1523/JNEUROSCI.19-15-06623.1999 10414990 PMC6782794

[B198] Quirk G. J. Gehlert D. R. (2003). Inhibition of the amygdala: key to pathological states? Ann. N. Y. Acad. Sci. 985, 263–272. 10.1111/j.1749-6632.2003.tb07087.x 12724164

[B199] Radley J. Morilak D. Viau V. Campeau S. (2015). Chronic stress and brain plasticity: mechanisms underlying adaptive and maladaptive changes and implications for stress-related CNS disorders. Neurosci. Biobehav. Rev. 58, 79–91. 10.1016/j.neubiorev.2015.06.018 26116544 PMC4684432

[B200] Resnik J. Paz R. (2015). Fear generalization in the primate amygdala. Nat. Neurosci. 18, 188–190. 10.1038/nn.3900 25531573

[B201] Rincón-Cortés M. Barr G. A. Mouly A. M. Shionoya K. Nuñez B. S. Sullivan R. M. (2015). Enduring good memories of infant trauma: rescue of adult neurobehavioral deficits via amygdala serotonin and corticosterone interaction. Proc. Natl. Acad. Sci. U. S. A. 112, 881–886. 10.1073/pnas.1416065112 25561533 PMC4311810

[B202] Robinson-Drummer P. A. Opendak M. Blomkvist A. Chan S. Tan S. Delmer C.. (2019). Infant trauma alters social buffering of threat learning: emerging role of prefrontal cortex in preadolescence. Front. Behav. Neurosci. 13:132. 10.3389/fnbeh.2019.00132 31293398 PMC6598593

[B203] Roth M. C. Humphreys K. L. King L. S. Gotlib I. H. (2018). Self-reported neglect, amygdala volume, and symptoms of anxiety in adolescent boys. Child Abuse Negl. 80, 80–89. 10.1016/j.chiabu.2018.03.016 29574295 PMC5953811

[B204] Rothbaum B. O. Price M. Jovanovic T. Norrholm S. D. Gerardi M. Dunlop B.. (2014). A randomized, double-blind evaluation of d -cycloserine or alprazolam combined with virtual reality exposure therapy for posttraumatic stress disorder in Iraq and Afghanistan War Veterans. Am. J. Psychiatry 171, 640–648. 10.1176/appi.ajp.2014.13121625 24743802 PMC4115813

[B205] Sanders S. K. Shekhar A. (1995). Regulation of anxiety by GABAA receptors in the rat amygdala. Pharmacol. Biochem. Behav. 52, 701–706. 10.1016/0091-3057(95)00153-N 8587908

[B206] Saul M. L. Helmreich D. L. Callahan L. M. Fudge J. L. (2014). Differences in amygdala cell proliferation between adolescent and young adult rats. Dev. Psychobiol. 56, 517–528. 10.1002/dev.21115 23775606

[B207] Saxbe D. Khoddam H. Piero L. D. Stoycos S. A. Gimbel S. I. Margolin G.. (2018). Community violence exposure in early adolescence: Longitudinal associations with hippocampal and amygdala volume and resting state connectivity. Dev. Sci. 21:e12686. 10.1111/desc.12686 29890029 PMC11694245

[B208] Schmahl C. G. Vermetten E. Elzinga B. M. Douglas Bremner J. (2003). Magnetic resonance imaging of hippocampal and amygdala volume in women with childhood abuse and borderline personality disorder. Psychiatry Res. Neuroimaging 122, 193–198. 10.1016/S0925-4927(03)00023-4 12694893

[B209] Schumann C. M. Amaral D. G. (2006). Stereological analysis of amygdala neuron number in autism. J. Neurosci. 26, 7674–7679. 10.1523/JNEUROSCI.1285-06.2006 16855095 PMC6674270

[B210] Shaw P. Greenstein D. Lerch J. Clasen L. Lenroot R. Gogtay N.. (2006). Intellectual ability and cortical development in children and adolescents. Nature 440, 676–679. 10.1038/nature04513 16572172

[B211] Shekhar A. Truitt W. Rainnie D. Sajdyk T. (2005). Role of stress, corticotrophin releasing factor (CRF) and amygdala plasticity in chronic anxiety. Stress 8, 209–219. 10.1080/10253890500504557 16423710

[B212] Sheline Y. I. Sanghavi M. Mintun M. A. Gado M. H. (1999). Depression duration but not age predicts hippocampal volume loss in medically healthy women with recurrent major depression. J. Neurosci. 19, 5034–5043. 10.1523/JNEUROSCI.19-12-05034.1999 10366636 PMC6782668

[B213] Sheridan M. A. Fox N. A. Zeanah C. H. McLaughlin K. A. Nelson C. A. (2012). Variation in neural development as a result of exposure to institutionalization early in childhood. Proc. Natl. Acad. Sci. U. S. A. 109, 12927–12932. 10.1073/pnas.1200041109 22826224 PMC3420193

[B214] Shonkoff J. P. Garner A. S. Siegel B. S. Dobbins M. I. Earls M. F. McGuinn L.. (2012). The lifelong effects of early childhood adversity and toxic stress. Pediatrics 129, e232–e246. 10.1542/peds.2011-2663 22201156

[B215] Sigal J. J. Perry J. C. Rossignol M. Ouimet M. C. (2003). Unwanted infants: psychological and physical consequences of inadequate orphanage care 50 years later. Am. J. Orthopsychiatry 73, 3–12. 10.1037/0002-9432.73.1.3 12674514

[B216] Slavich G. M. (2019). Stressnology: the primitive (and problematic) study of life stress exposure and pressing need for better measurement. Brain. Behav. Immun. 75, 3–5. 10.1016/j.bbi.2018.08.011 30236597 PMC6279572

[B217] Smith K. E. Pollak S. D. (2020). Rethinking concepts and categories for understanding the neurodevelopmental effects of childhood adversity. Perspect. Psychol. Sci. 2020:1745691620920725. 10.1177/1745691620920725 32668190 PMC7809338

[B218] Soeter M. Kindt M. (2015). An abrupt transformation of phobic behavior after a post-retrieval amnesic agent. Biol. Psychiatry 78, 880–886. 10.1016/j.biopsych.2015.04.006 25980916

[B219] Sorrells S. F. Paredes M. F. Velmeshev D. Herranz-Pérez V. Sandoval K. Mayer S.. (2019). Immature excitatory neurons develop during adolescence in the human amygdala. Nat. Commun. 10:2748. 10.1038/s41467-019-10765-1 31227709 PMC6588589

[B220] Southwick S. M. Bonanno G. A. Masten A. S. Panter-Brick C. Yehuda R. (2014). Resilience definitions, theory, and challenges: interdisciplinary perspectives. Eur. J. Psychotraumatol. 5:25338. 10.3402/ejpt.v5.25338 25317257 PMC4185134

[B221] Souza-Queiroz J. Boisgontier J. Etain B. Poupon C. Duclap D. d'Albis M.-A.. (2016). Childhood trauma and the limbic network: a multimodal MRI study in patients with bipolar disorder and controls. J. Affect. Disord. 200, 159–164. 10.1016/j.jad.2016.04.038 27136413

[B222] Steptoe A. Feldman P. J. (2001). Neighborhood problems as sources of chronic stress: development of a measure of neighborhood problems, and associations with socioeconomic status and health. Ann. Behav. Med. 23, 177–185. 10.1207/S15324796ABM2303_5 11495218

[B223] Strobel C. Marek R. Gooch H. M. Sullivan R. K. P. Sah P. (2015). Prefrontal and auditory input to intercalated neurons of the amygdala. Cell Rep. 10, 1435–1442. 10.1016/j.celrep.2015.02.008 25753409

[B224] Suor J. H. Jimmy J. Monk C. S. Phan K. L. Burkhouse K. L. (2020). Parsing differences in amygdala volume among individuals with and without social and generalized anxiety disorders across the lifespan. J. Psychiatr. Res. 128, 83–89. 10.1016/j.jpsychires.2020.05.027 32544774 PMC7483375

[B225] Sutoo D. E. Akiyama K. Yabe K. (2000). Quantitative maps of GABAergic and glutamatergic neuronal systems in the human brain. Hum. Brain Mapp. 11, 93–103. 11061336 10.1002/1097-0193(200010)11:2<93::AID-HBM30>3.0.CO;2-Y PMC6872118

[B226] Suvrathan A. Bennur S. Ghosh S. Tomar A. Anilkumar S. Chattarji S. (2014). Stress enhances fear by forming new synapses with greater capacity for long-term potentiation in the amygdala. Philos. Trans. R. Soc. B Biol. Sci. 369:20130151. 10.1098/rstb.2013.0151 24298153 PMC3843883

[B227] Tarrier N. Sommerfield C. Pilgrim H. (1999). Relatives' expressed emotion (EE) and PTSD treatment outcome. Psychol. Med. 29, 801–811. 10.1017/S0033291799008569 10473307

[B228] Taylor P. J. Gooding P. Wood A. M. Tarrier N. (2011). The role of defeat and entrapment in depression, anxiety, and suicide. Psychol. Bull. 137, 391–420. 10.1037/a0022935 21443319

[B229] Tedeschi R. G. Calhoun L. G. (2004). TARGET ARTICLE: “Posttraumatic growth: conceptual foundations and empirical evidence.” Psychol. Inq. 15, 1–18. 10.1207/s15327965pli1501_01

[B230] Teisl M. Cicchetti D. (2008). Physical abuse, cognitive and emotional processes, and aggressive/disruptive behavior problems. Soc. Dev. 17, 1–23. 10.1111/j.1467-9507.2007.00412.x

[B231] Telch M. J. Bruchey A. K. Rosenfield D. Cobb A. R. Smits J. Pahl S.. (2014). Effects of post-session administration of methylene blue on fear extinction and contextual memory in adults with claustrophobia. Am. J. Psychiatry 171, 1091–1098. 10.1176/appi.ajp.2014.13101407 25018057 PMC4467026

[B232] Tieman W. van der Ende J. Verhulst F. C. (2005). Psychiatric disorders in young adult intercountry adoptees: an epidemiological study. Am. J. Psychiatry 162, 592–598. 10.1176/appi.ajp.162.3.592 15741478

[B233] Tottenham N. Hare T. A. Quinn B. T. McCarry T. W. Nurse M. Gilhooly T.. (2010). Prolonged institutional rearing is associated with atypically large amygdala volume and difficulties in emotion regulation. Dev. Sci. 13, 46–61. 10.1111/j.1467-7687.2009.00852.x 20121862 PMC2817950

[B234] Tromp D. P. M. Fox A. S. Oler J. A. Alexander A. L. Kalin N. H. (2019). The relationship between the uncinate fasciculus and anxious temperament is evolutionarily conserved and sexually dimorphic. Biol. Psychiatry 86, 890–898. 10.1016/j.biopsych.2019.07.022 31542153 PMC6910082

[B235] Tye K. M. Prakash R. Kim S.-Y. Fenno L. E. Grosenick L. Zarabi H.. (2011). Amygdala circuitry mediating reversible and bidirectional control of anxiety. Nature 471, 358–362. 10.1038/nature09820 21389985 PMC3154022

[B236] Tzanoulinou S. García-Mompó C. Castillo-Gómez E. Veenit V. Nacher J. Sandi C. (2014a). Long-term behavioral programming induced by peripuberty stress in rats is accompanied by GABAergic-related alterations in the amygdala. PLoS ONE 9:e94666. 10.1371/journal.pone.0094666 24736324 PMC3988094

[B237] Tzanoulinou S. Riccio O. de Boer M. W. Sandi C. (2014b). Peripubertal stress-induced behavioral changes are associated with altered expression of genes involved in excitation and inhibition in the amygdala. Transl. Psychiatry 4:e410. 10.1038/tp.2014.54 25004390 PMC4119221

[B238] Ulfig N. Setzer M. Bohl J. (2003). Ontogeny of the human amygdala. Ann. N. Y. Acad. Sci. 985, 22–33. 10.1111/j.1749-6632.2003.tb07068.x 12724145

[B239] Uno H. Tarara R. Else J. G. Suleman M. A. Sapolsky R. M. (1989). Hippocampal damage associated with prolonged and fatal stress in primates. J. Neurosci. 9, 1705–1711. 10.1523/JNEUROSCI.09-05-01705.1989 2723746 PMC6569823

[B240] VanTieghem M. Korom M. Flannery J. Choy T. Caldera C. Humphreys K. L.. (2021). Longitudinal changes in amygdala, hippocampus and cortisol development following early caregiving adversity. Dev. Cognit. Neurosci. 48:100916. 10.1016/j.dcn.2021.100916 33517107 PMC7848778

[B241] Veer I. M. Oei N. Y. L. van Buchem M. A. Spinhoven P. Elzinga B. M. Rombouts S. A. R. B. (2015). Evidence for smaller right amygdala volumes in posttraumatic stress disorder following childhood trauma. Psychiatry Res. Neuroimaging 233, 436–442. 10.1016/j.pscychresns.2015.07.016 26211620

[B242] Velakoulis D. Wood S. J. Wong M. T. H. McGorry P. D. Yung A. Phillips L.. (2006). Hippocampal and amygdala volumes according to psychosis stage and diagnosis: a magnetic resonance imaging study of chronic schizophrenia, first-episode psychosis, and ultra–high-risk individuals. Arch. Gen. Psychiatry 63:139. 10.1001/archpsyc.63.2.139 16461856

[B243] Votruba-Drzal E. (2006). Economic disparities in middle childhood development: does income matter? Dev. Psychol. 42, 1154–1167. 10.1037/0012-1649.42.6.1154 17087549

[B244] Vyas A. Bernal S. Chattarji S. (2003). Effects of chronic stress on dendritic arborization in the central and extended amygdala. Brain Res. 965, 290–294. 10.1016/S0006-8993(02)04162-8 12591150

[B245] Vyas A. Jadhav S. Chattarji S. (2006). Prolonged behavioral stress enhances synaptic connectivity in the basolateral amygdala. Neuroscience 143, 387–393. 10.1016/j.neuroscience.2006.08.003 16962717

[B246] Vyas A. Mitra R. Rao B. S. Chattarji S. (2002). Chronic stress induces contrasting patterns of dendritic remodeling in hippocampal and amygdaloid neurons. J. Neurosci. 22, 6810–6818. 10.1523/JNEUROSCI.22-15-06810.2002 12151561 PMC6758130

[B247] Vyas A. Pillai A. G. Chattarji S. (2004). Recovery after chronic stress fails to reverse amygdaloid neuronal hypertrophy and enhanced anxiety-like behavior. Neuroscience 128, 667–673. 10.1016/j.neuroscience.2004.07.013 15464275

[B248] Wang X. Liu H. Morstein J. Novak A. J. E. Trauner D. Xiong Q.. (2020). Metabolic tuning of inhibition regulates hippocampal neurogenesis in the adult brain. Proc. Natl. Acad. Sci. U.S.A. 117, 25818–25829. 10.1073/pnas.2006138117 32973092 PMC7568294

[B249] Watanabe Y. Gould E. McEwen B. S. (1992). Stress induces atrophy of apical dendrites of hippocampal CA3 pyramidal neurons. Brain Res. 588, 341–345. 10.1016/0006-8993(92)91597-8 1393587

[B250] Weiner A. Kupermintz H. (2001). Facing adulthood alone: the long-term impact of family break-up and infant institutions, a longitudinal study. Br. J. Soc. Work 31, 213–234. 10.1093/bjsw/31.2.213

[B251] Weintraub M. J. Hall D. L. Carbonella J. Y. Weisman de Mamani A. Hooley J. M. (2017). Integrity of literature on expressed emotion and relapse in patients with schizophrenia verified by ap-curve analysis. Fam. Process 56, 436–444. 10.1111/famp.12208 26875506 PMC5765756

[B252] Weissman D. G. Lambert H. K. Rodman A. M. Peverill M. Sheridan M. A. McLaughlin K. A. (2020). Reduced hippocampal and amygdala volume as a mechanism underlying stress sensitization to depression following childhood trauma. Depress. Anxiety 37, 916–925. 10.1002/da.23062 32579793 PMC7484449

[B253] Weniger G. Lange C. Sachsse U. Irle E. (2008). Amygdala and hippocampal volumes and cognition in adult survivors of childhood abuse with dissociative disorders. Acta Psychiatr. Scand. 118, 281–290. 10.1111/j.1600-0447.2008.01246.x 18759808

[B254] Whittle S. Dennison M. Vijayakumar N. Simmons J. G. Yücel M. Lubman D. I.. (2013). Childhood maltreatment and psychopathology affect brain development during adolescence. J. Am. Acad. Child Adolesc. Psychiatry 52, 940–952. 10.1016/j.jaac.2013.06.007 23972696

[B255] Wierenga L. M. Langen M. Oranje B. Durston S. (2014). Unique developmental trajectories of cortical thickness and surface area. NeuroImage 87, 120–126. 10.1016/j.neuroimage.2013.11.010 24246495

[B256] Wiersma J. E. Hovens J. G. F. M. Van Oppen P. Giltay E. J. Van Schaik D. J. F. Beekman A. T. F.. (2009). The importance of childhood trauma and childhood life events for chronicity of depression in adults. J. Clin. Psychiatry 70, 983–989. 10.4088/JCP.08m04521 19653975

[B257] Williams L. M. Debattista C. Duchemin A. M. Schatzberg A. F. Nemeroff C. B. (2016). Childhood trauma predicts antidepressant response in adults with major depression: data from the randomized international study to predict optimized treatment for depression. Transl. Psychiatry 6, e799–e797. 10.1038/tp.2016.61 27138798 PMC5070060

[B258] Wilson H. W. Stover C. S. Berkowitz S. J. (2009). Research Review: the relationship between childhood violence exposure and juvenile antisocial behavior: a meta-analytic review. J. Child Psychol. Psychiatry 50, 769–779. 10.1111/j.1469-7610.2008.01974.x 19017367

[B259] Woon F. L. Hedges D. W. (2009). Amygdala volume in adults with posttraumatic stress disorder: a meta-analysis. J. Neuropsychiatry. 21, 5–12. 10.1176/jnp.2009.21.1.5 19359446

[B260] Zhang H. Schneider T. Wheeler-Kingshott C. A. Alexander D. C. (2012). NODDI: practical *in vivo* neurite orientation dispersion and density imaging of the human brain. NeuroImage 61, 1000–1016. 10.1016/j.neuroimage.2012.03.072 22484410

[B261] Zhang W. Rosenkranz J. A. (2012). Repeated restraint stress increases basolateral amygdala neuronal activity in an age-dependent manner. Neuroscience 226, 459–474. 10.1016/j.neuroscience.2012.08.051 22986163 PMC3506707

[B262] Zhang X. Kim J. Tonegawa S. (2020). Amygdala reward neurons form and store fear extinction memory. Neuron 105, 1077–1093.e7. 10.1016/j.neuron.2019.12.025 31952856

[B263] Zoellner L. A. Telch M. Foa E. B. Farach F. J. McLean C. P. Gallop R.. (2017). Enhancing extinction learning in posttraumatic stress disorder with brief daily imaginal exposure and methylene blue: a randomized controlled trial. J. Clin. Psychiatry 78, e782–e789. 10.4088/JCP.16m10936 28686823

